# Errors in soil maps: The need for better on-site estimates and soil map predictions

**DOI:** 10.1371/journal.pone.0270176

**Published:** 2023-01-11

**Authors:** Michaela Buenemann, Marina E. Coetzee, Josephat Kutuahupira, Jonathan J. Maynard, Jeffrey E. Herrick

**Affiliations:** 1 Department of Geography, New Mexico State University, Las Cruces, New Mexico, United States of America; 2 Faculty of Natural Resources and Spatial Sciences, Namibia University of Science and Technology, Windhoek, Namibia; 3 Division of Plant Production, Ministry of Agriculture, Water, and Forestry, Windhoek, Namibia; 4 Sustainability Innovation Lab, University of Colorado, Boulder, Colorado, United States of America; 5 Jornada Experimental Range, Agricultural Research Service, United States Department of Agriculture, Las Cruces, New Mexico, United States of America; Indiana State University, UNITED STATES

## Abstract

High-quality soil maps are urgently needed by diverse stakeholders, but errors in existing soil maps are often unknown, particularly in countries with limited soil surveys. To address this issue, we used field soil data to assess the accuracy of seven spatial soil databases (Digital Soil Map of the World, Namibian Soil and Terrain Digital Database, Soil and Terrain Database for Southern Africa, Harmonized World Soil Database, SoilGrids1km, SoilGrids250m, and World Inventory of Soil Property Estimates) using topsoil texture as an example soil property and Namibia as a case study area. In addition, we visually compared topsoil texture maps derived from these databases. We found that the maps showed the correct topsoil texture in only 13% to 42% of all test sites, with substantial confusion occurring among all texture categories, not just those in close proximity in the soil texture triangle. Visual comparisons of the maps moreover showed that the maps differ greatly with respect to the number, types, and spatial distribution of texture classes. The topsoil texture information provided by the maps is thus sufficiently inaccurate that it would result in significant errors in a number of applications, including irrigation system design and predictions of potential forage and crop productivity, water runoff, and soil erosion. Clearly, the use of these existing maps for policy- and decision-making is highly questionable and there is a critical need for better on-site estimates and soil map predictions. We propose that mobile apps, citizen science, and crowdsourcing can help meet this need.

## Introduction

High-quality soil maps are indispensable for achieving sustainable socio-ecological systems, but errors in existing soil maps (i.e., differences between mapped soil properties and reference or true soil properties as determined in the laboratory) are often unknown, making their use for natural resources management and modeling questionable in many cases. This problem is further exacerbated by the fact that map users frequently are not given adequate information (e.g., spatial resolution and map accuracy) to properly judge the suitability of a map for a particular purpose. For example, a world soil map with a spatial resolution of 1 km may be valuable for modeling carbon sequestration at the global scale, but not for making irrigation decisions at the farm scale. The lack of such critical metadata accompanying soil maps is so pervasive that some researchers have developed map assessment guidelines for soil map users [e.g., 1]. High-quality soil maps are urgently needed by a variety of stakeholders for many reasons. Soil data are necessary to answer questions related to issues such as agricultural and other land management, land use planning, human health, cultural heritage, archaeological and paleoenvironmental reconstructions, and natural hazards [e.g., 2]. Soil knowledge is also critical to better understand the supporting, regulating, provisioning, and cultural ecosystem services provided by soils and, hence, to find ways to enhance human well-being [3, 4]. Given accelerated soil degradation and continued human pressures on soil in the Anthropocene [5–7], better soil information is required if we are to maintain or improve soil security, which plays an indispensable role in the global environmental sustainability challenges of climate stability, biodiversity, food security, water security, energy security, and ecosystem service delivery [8–10]. Stakeholders in soil data thus include farmers and ranchers, land use planners, environmental extension agents, scientists, and policy-makers.

While the need for high-quality spatial soil data is increasingly recognized [2], existing soil maps fall short of the expectations of such data. High-quality soil data may be defined as soil data that are up-to-date, sufficiently accurate for their intended purpose, associated with uncertainty information, three-dimensional, available at multiple spatial resolutions, spatio-temporally explicit and continuous, affordable, easily integrated with other digital spatial data, and readily available for interested stakeholders [5, 7, 11]. In contrast, existing soil data are criticized for being outdated, having a low spatial resolution, missing data in some regions, suffering from logical inconsistencies, and, importantly, for lacking information about errors and uncertainties [2, 12].

Despite these criticisms, soil maps of various spatial resolutions, geographic extents, and soil characteristics have been used in numerous application contexts for many objectives. To give just a few examples, three soil attributes (organic matter content, soil layer depth, bulk density) from the U.S. Soil Survey Geographic Database (1:12,000 or 1:24,000) and the U.S. State Soil Geographic Database (1:250,000) were used to estimate soil organic carbon in the U.S. state of Louisiana [13]. Five attributes (type, surface rockiness, surface stoniness, degree of erosion, rooting depth) from the Soil and Terrain Database for Southern Africa (1:2,000,000) were used to explore the influence of soils on early summer vegetative activity in north-central South Africa [14]. Eight attributes of the dominant topsoil (textural class, coarse fragment volume, gypsum content, base saturation content, pH, organic carbon content, salinity, sodicity) from the International Soil Reference and Information Centre’s World Inventory of Soil property Estimates (5 × 5 arc-minutes) and the Harmonized World Soil Database (30 × 30 arc-seconds) were used to determine site suitability for crop growth across the world [15]. The Soil Map of the World (1:5,000,000) has been used to assess desertification, delineate agro-ecological zones, calculate population supporting capacity, and many other applications [16].

Because errors and uncertainties in most of these maps are not defined, the reliability of the derived estimates is poorly understood. Furthermore, there have been relatively few assessments of the variability among currently available soil maps. The main objective of this paper is to address these issues by comparing and evaluating errors in seven topsoil texture maps of Namibia (Digital Soil Map of the World, Namibian Soil and Terrain Digital Database, Soil and Terrain Database for Southern Africa, Harmonized World Soil Database, SoilGrids1km, SoilGrids250m, and World Inventory of Soil Property Estimates). Soil surface texture was selected for the comparisons and evaluations because it influences nearly all physical, chemical, and biological soil processes [17] and, hence, the soil’s potential for providing ecosystem services and human well-being [4]. Namibia was selected as an example country because it is representative of many countries in which a) the majority of the population depends directly or indirectly on its soil capital, b) multiple soil maps are available, and c) users of soil maps have little information available to evaluate or improve the accuracy and precision of soil predictions based on these maps. Namibia was also selected because it has a large, national-level soil profile dataset (over 1,000 topsoil textures) suitable for soil map evaluation.

## Materials and methods

### Study area

**Fig 1:**
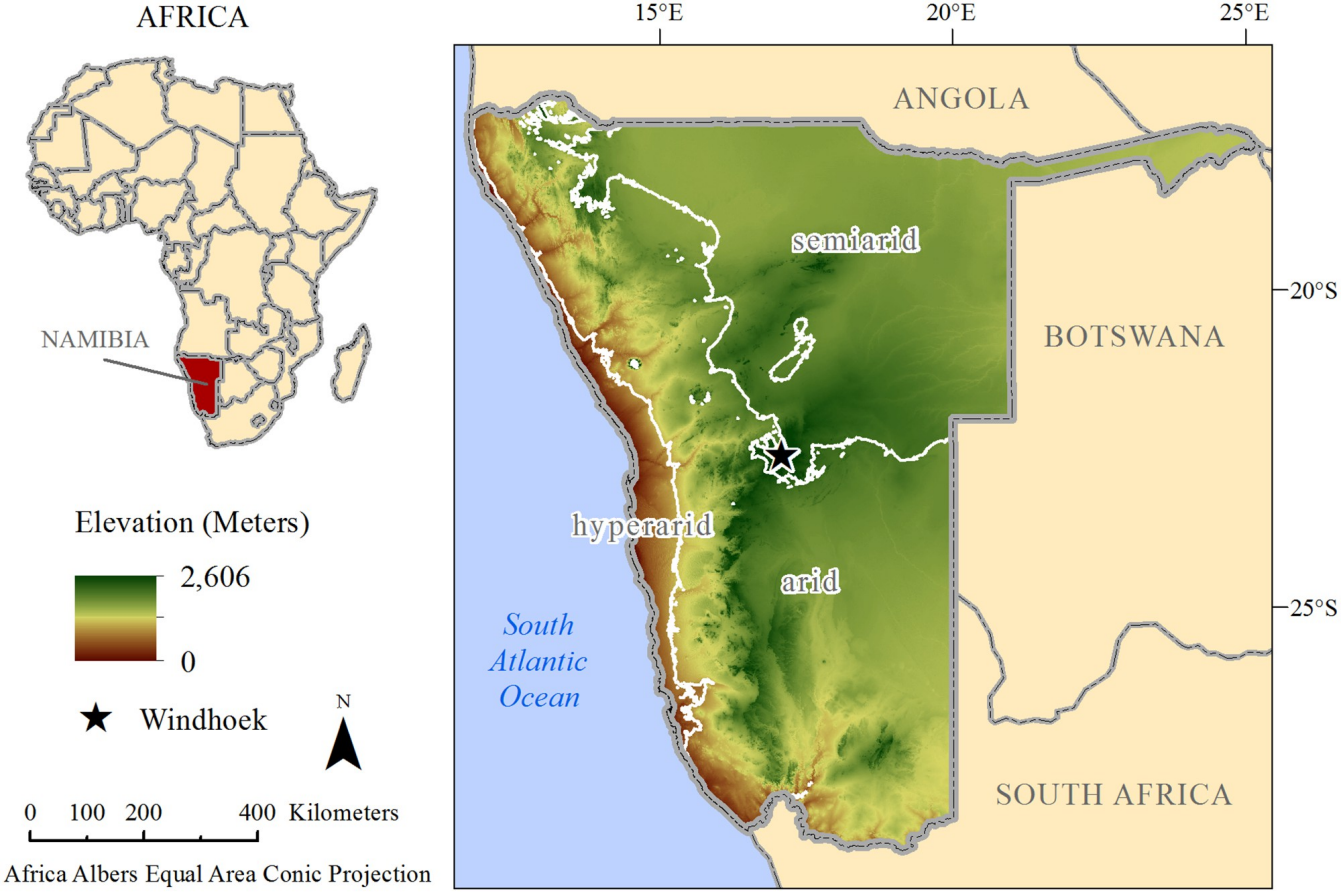
Location and topo-climatic characteristics of Namibia. White lines indicate borders between three aridity zones: semiarid (aridity index ~ 0.2 to 0.371), arid (aridity index ~ 0.03 to 0.2), and hyperarid (aridity index ~ 0.006 to 0.03).

Namibia is an environmentally diverse country in which soils are crucial for human well-being. Located in southwestern Africa, Namibia extends from about 17° S to 29° S and 11.7° E to 25.3° E and encompasses an area of about 825,615 km^2^ (Fig 1). The country is characterized primarily by hot desert (BWh) and hot steppe (BSh) climates [18], with aridity generally increasing from the subhumid to semiarid northeast through the arid center to the hyperarid southwest [19]. Elevations in Namibia range from 0 m along the Atlantic Coast to 2,606 m at the Königstein in the Brandberg Massif [20]. Landscapes and landforms in the country are extremely diverse, encompassing plains, plateaus, inselbergs, canyons, mountains, pans, dunes, and more [20]. Six major biomes occur in Namibia, including Namib desert, succulent karoo, Nama karoo, *Acacia* tree and shrub savanna, broadleaved tree and shrub savanna, and lakes and salt pans [21]. Soils vary markedly across the country as a result of variations in climate, topography, geology, and biology. Major soil groups include Arenosols, Leptosols, Calcisols, Gypsisols, Regosols, and Cambisols [22]. Soils are of utmost importance to Namibia as the majority of the population depends directly on the natural resources that soils support, including and not limited to wood for building homes and cooking food; crops for food, feed, fuel, and fiber; and graze and browse for livestock [21]. Given this significance of soils in determining the land’s potential to support livelihoods in Namibia, and given the country’s ongoing issues of desertification, land degradation, and drought [23], accurate and up-to-date soil information is vital for identifying sustainable land management strategies in Namibia.

### Soil map and field data acquisition and processing

We used two major groups of data in this research: soil map data products with topsoil texture information and field soil data for assessing the accuracy of existing topsoil texture map products.

**Fig 2:**
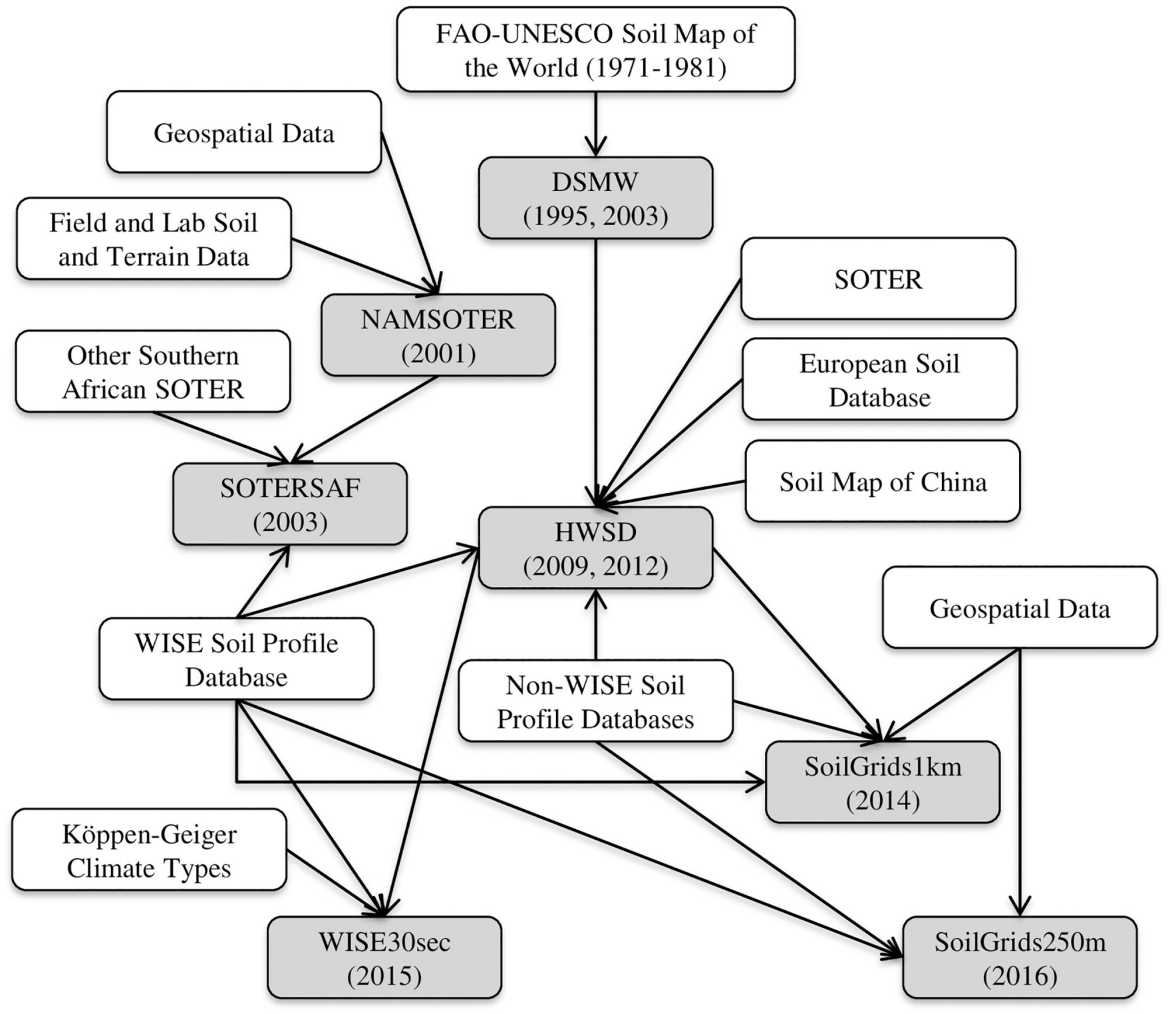
Relationships between the seven soil databases used in this study. The following databases were considered: DSMW, NAMSOTER, SOTERSAF, HWSD, SoilGrids1km, SoilGrids250m, and WISE30sec. See the text for full names and versions of the soil databases. “Field and Lab Soil and Terrain Data” are based on data collected at discrete points on the ground; “Geospatial Data” are derived mostly from remotely sensed products and cover areas as opposed to points.

The soil map data were derived from seven different products (Table 1; see S1 Text for a more detailed description of each): the Digital Soil Map of the World (DSMW, Version 3.6), the NAMibian SOil and TERrain Digital Database (NAMSOTER, Version 1.0), the SOil and TERrain Database for Southern AFrica (SOTERSAF, Version 1.0), the Harmonized World Soil Database (HWSD, Version 1.21), SoilGrids1km (Version 1.0), SoilGrids250m (Version 1.0), and the World Inventory of Soil property Estimates (WISE30sec, Version 1.0). While the seven products were generated by different agencies using different data and methods at different times and different spatial scales, several of the products are related to each other (Fig 2). Broadly speaking, five of the maps were created using conventional soil mapping (DSMW, NAMSOTER, SOTERSAF, HWSD, WISE30sec) and two using digital soil mapping (SoilGrids1km, SoilGrids250m). Conventional soil mapping is a largely based on the manual delineation of soil map units and the linking of these units with soil attributes using expert knowledge. In contrast, digital soil mapping is a largely automated, quantitative approach that entails the spatially explicit and continuous prediction of soil properties through models that link soil observations at points in the field and geospatial layers representing climate, vegetation, topography, and other factors [24, 25].

**Table 1:** Key characteristics of topsoil texture map data sources.

Name^a^ (Type)	Publication Year (Version)	Agency^b^	Spatial Detail	Spatial Extent	Production Method	References
DSMW (Vector)	1995 (v. 3.5) 2003 (v. 3.6)	FAO	1:5,000,000^c^	World	Digitizing, linking of map units with attribute data	[26–29]
NAMSOTER (Vector)	2001 (v.1.0)	MAWRD	1:1,000,000^c^	Namibia	Digitizing, linking of map units with attribute data	[22]
SOTERSAF (Vector)	2003 (v. 1.0)	ISRIC, FAO, UNEP, National Soil Institutes	1:2,000,000^c^	Southern Africa	Merging of existing soil map units, linking of map units with attribute data	[30–32]
HWSD (Raster)	2009 (v. 1.1) 2012 (v. 1.21)	FAO, IIASA, ISRIC, ESBN-JRC, ISS-CAS	30 × 30 arc-seconds^d^	World	Merging of existing soil map units, linking of map units with attribute data	[33–36]
SoilGrids1km (Raster)	2013 (v. 1.0)	ISRIC	1 × 1 km^d^	World	Regression	[37, 38]
SoilGrids250m (Raster)	2016 (v. 1.0^e^)	ISRIC	250 × 250 m^d^	World	Machine learning	[39, 40]
WISE30sec (Raster)	2015 (v. 1.0)	ISRIC	30 × 30 arc-seconds^d^	World	Overlay of existing soil and climate maps; linking of map units with attribute data	[41–43]

^a^ See text for full names.

^b^ FAO = Food and Agriculture Organization of the United Nations, MAWRD = Namibia Ministry of Agriculture, Water, and Rural Development (now MAWF = Namibia Ministry of Agriculture, Water, and Forestry, ISRIC = International Soil Reference and Information Centre, UNEP = United Nations Environment Programme, IIASA = International Institute for Applied Systems Analysis, ESBN-JRC = European Soil Bureau Network Joint Research Centre, ISS-CAS = Institute of Soil Science Chinese Academy of Sciences.

^c^ Stated map scale of vector data; map scales of 1:1,000,000, 1:2,000,000, and 1:5,000,000 translate to spatial resolutions of roughly 500 m, 1 km, and 2.5 km, respectively.

^d^ Stated spatial resolution of raster data; a spatial resolution of 30 arc-seconds translates to a spatial resolution of roughly 1 km.

^e^ This is the first global SoilGrids250m product; an earlier SoilGrids250m dataset is available for Africa [44, 45].

**Fig 3:**
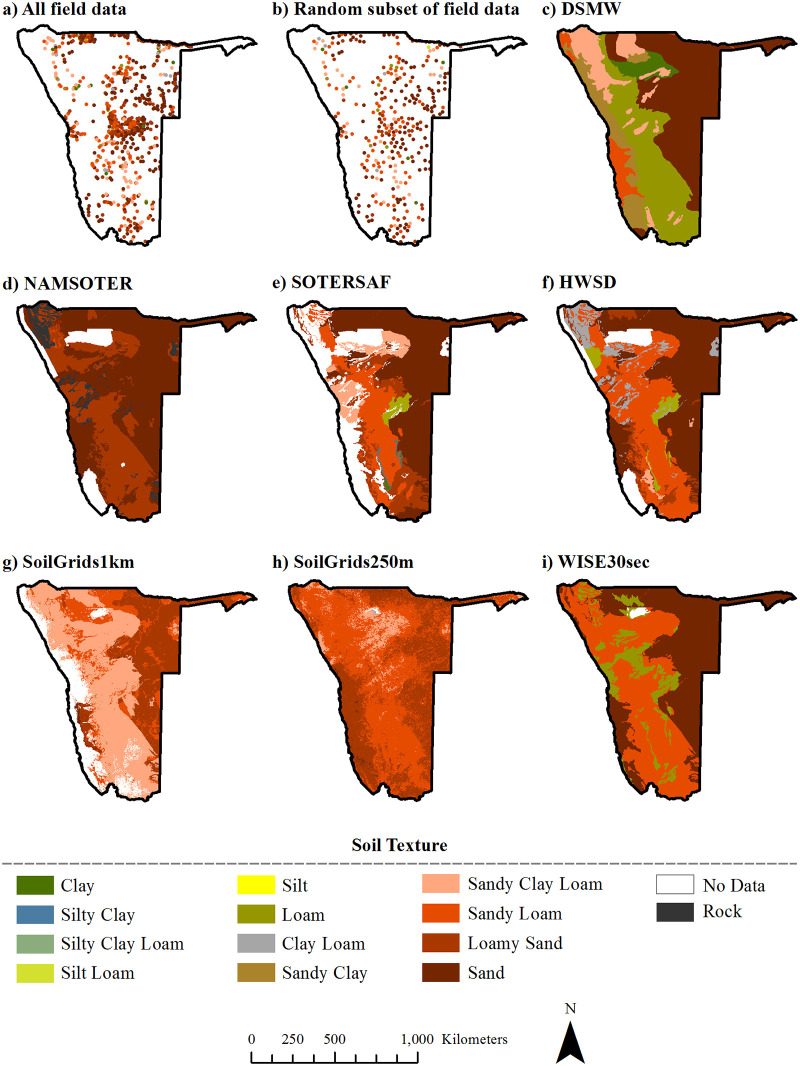
Dominant topsoil textures in Namibia according to the soil databases used in this study. The panels show: a) full field dataset (1,102 samples), b) spatially uncorrelated subset of field data 327 samples), c) DSMW, d) NAMSOTER, e) SOTERSAF, f) HWSD, g) SoilGrids1km, h) SoilGrids250m, and i) WISE30sec. See the text for the full names and versions of the databases.

Map topsoil texture data were obtained from the products in several steps. We acquired six of the seven soil datasets from online repositories: DSMW came from an FAO website [27], HWSD from an International Institute for Applied Systems Analysis (IIASA) website [34], and SOTERSAF, ISRIC-WISE, SoilGrids1km, and SoilGrids250 m from websites of the International Soil Reference and Information Centre [ISRIC; 30, 37, and 40, respectively, 46]. NAMSOTER data were obtained from the MAWF [22]. Once acquired, we preprocessed all datasets to share the same spatial reference system (Namibia Albers Equal Area) and geographic extent (Namibia). Following the preprocessing, we extracted United States Department of Agriculture (USDA) soil textural class information for all map units in the vector maps (DSMW, NAMSOTER, SOTERSAF) and for all pixels in the raster maps (HWSD, WISE30sec, SoilGrids1km, SoilGrids250m). In NAMSOTER, SOTERSAF, and HWSD textural classes were directly included as a database attribute for depths of 0–20 cm, 0–20 cm, and 0–30 cm. SoilGrids250m also directly provided texture class information, but at depths of 0, 5, 15, and 30 cm; in this case, we extracted the texture class that was dominant across the four depths. For DSMW, SoilGrids1km, and WISE30sec, we calculated USDA textural classes based on sand, silt, and clay fractions given in the databases for depths of 0–20 cm, 0–30 cm, and 0–20 cm, respectively. The final products included topsoil texture maps based on the DSMW (Fig 3c), NAMSOTER (Fig 3d), SOTERSAF (Fig 3e), the HWSD (Fig 3f), SoilGrids1km (Fig 3g), SoilGrids250m (Fig 3h), and WISE30sec (Fig 3i).

The field soil data were collected as part of the National Soil Survey of Namibia, one of several projects of Namibia’s Agro-Ecological Zoning (AEZ) Programme [47]. The survey was conducted jointly by Spain’s Cartographic Institute of Catalonia (ICC) and the AEZ team of Namibia’s Ministry of Agriculture, Water, and Rural Development (MAWRD) between 1998 and 2000 and subsequently by the AEZ team only. Data were collected from 1,102 locations across Namibia, most notably in central Namibia near the capital Windhoek and in northern Namibia where agricultural production potential is highest in the country. The data were described in the field according to guidelines of the Food and Agriculture Organization of the United Nations [FAO; 48] and analyzed in the Agricultural Laboratory of the MAWRD following its standard operating procedures [49–51]. The texture of topsoil samples relevant here was reported in terms of both sand, silt, and clay fractions and USDA textural classes [52]. We acquired the field data from Namibia’s Ministry of Agriculture, Water, and Forestry (MAWF; formerly MAWRD) (Fig 3a, Table 2) and extracted topsoil texture data for each of the 1,102 field locations to serve as potential reference data against which to compare the soil map data. Most of the field reference data were spatially autocorrelated, an issue known to lead to overoptimistic map accuracy results [53]. To avoid inflation of map accuracy results, we randomly extracted as many field samples as possible with a minimum distance of 20 km between sites—at that distance, the spatial distribution of samples was random according to average nearest neighbor statistics. This process resulted in a total of 327 spatially uncorrelated field samples (Fig 3b, Table 2, see S1 Dataset for the field soil data), which we then used to assess the accuracy of the spatial data described below (i.e., all subsequent results and discussions involving field data are based on the reduced spatially uncorrelated dataset and not on the original full spatially autocorrelated dataset).

**Table 2:** Allocation of field samples among soil textural classes in the full field data set and the spatially uncorrelated random subset of field data.

Lab Texture	All Field Data		Random Subset of Field Data	
	Number	Percent	Number	Percent
**Clay**	11	1.0	5	1.5
**Silty Clay**	0	0.0	0	0.0
**Silty Clay Loam**	0	0.0	0	0.0
**Silt Loam**	6	0.5	1	0.3
**Silt**	0	0.0	0	0.0
**Loam**	16	1.5	5	1.5
**Clay Loam**	20	1.8	5	1.5
**Sandy Clay**	10	0.9	2	0.6
**Sandy Clay Loam**	91	8.3	35	10.7
**Sandy Loam**	213	19.3	78	23.9
**Loamy Sand**	262	23.8	62	19.0
**Sand**	473	42.9	134	41.0
**Sum**	1,102	100.0	327	100.0

### Topsoil texture map accuracy assessment

We assessed the accuracy of the seven maps using error matrices and several map- and category-level accuracy measures [54], as described in detail below. An initial observed sample error matrix for each of the maps was constructed by cross-tabulating map topsoil texture values (rows) against field topsoil texture values (columns). The diagonal cells in this matrix summarize the sample units that were assigned to the correct category; the off-diagonal cells summarize every error in the map relative to the ground reference information and thus provide information on omission and commission errors. Because field reference data were not available for the entire study area and also not proportional to the size of the different topsoil texture strata in the different maps, the observed sample error matrices give biased information concerning the relationships between map and reference data. To address this issue, we converted all observed biased sample error matrices into “estimated unbiased population” error matrices (sensu [54]) using Eq 1 [55]:

$$p_{ij}=\left(\frac{n_{ij}}{n_{i+}}\right)\left(\frac{A_{m,i}}{A_{tot}}\right)$$ (1)

where *p*_*ij*_ is the estimated proportion of the study area that is topsoil texture category *i* according to the map data and topsoil texture category *j* according to the field data, where *i*, *j* = 1, …, *c*; *n*_*ij*_ is entry *n*_*ij*_ in row *i* and column *j* of the sample matrix; *n*_*i+*_ is the row total of category *i* in the sample matrix; *A*_*m*,*i*_ is the mapped area of category *i*, and *A*_*tot*_ is the total area of the map (Note: The “estimated unbiased population” error matrices may still contain some bias. However, without an additional completely random sample, this would be impossible to address. Additionally, such a sample would create other problems, because it would ignore spatial structure).

Subsequently, we derived the following category-level accuracy measures from each of the estimated error matrices, where *p*_*i+*_ and *p*_*+i*_ are the row and column totals of category *i*, respectively; υ_*i*_ is the user’s accuracy (Eq 2), π_*i*_ is the producer’s accuracy (Eq 3), *q*_*i*_ is the absolute quantity disagreement (Eq 4), *a*_*i*_ is the absolute allocation disagreement (Eq 5), *d*_*i*_ is the absolute general disagreement (Eq 6), *ϕ*_*i*_ is the relative quantity disagreement (Eq 7), *α*_*i*_ is the relative allocation disagreement (Eq 8), and *δ*_*i*_ is the relative general disagreement (Eq 9) for topsoil texture category *i* [54, 56, 57]:

$$\upsilon_{i}=\frac{p_{ii}}{p_{i+}}$$ (2)

$$\pi_{i}=\frac{p_{ii}}{p_{+i}}$$ (3)

$$q_{i}=\left|p_{i+}-p_{+i}\right|$$ (4)

$$a_{i}=2\text{min}\left(p_{i+}-p_{ii},p_{+i}-p_{ii}\right)$$ (5)

$$d_{i}=q_{i}+a_{i}$$ (6)

$$\phi_{i}=\frac{q_{i}}{p_{i+}+p_{+i}}$$ (7)

$$\alpha_{i}=\frac{a_{i}}{p_{i+}+p_{+i}}$$ (8)

$$\delta_{i}=\phi_{i}+\alpha_{i}$$ (9)

User’s accuracy indicates the probability that the category shown in a particular location on the map actually occurs in that location on the ground; it is the complement of the probability of commission error. Producer’s accuracy indicates the probability that the category observed in a particular location on the ground is actually shown in that location on the map; it is the complement of the probability of omission error. Absolute quantity disagreement describes the amount of difference between the field and map data resulting from the less than ideal match in the proportions of the topsoil texture categories. Absolute allocation disagreement describes the amount of difference between the field and map data that resulting from the less than ideal match in the spatial allocation of the topsoil texture categories, given the proportions of these classes in the field and map data. Absolute general disagreement is the sum of absolute quantity and allocation disagreement. Relative quantity, allocation, and general disagreement measure the same information as absolute quantity, allocation, and general disagreement, respectively, but take into account the abundance of category *i*.

Finally, we derived the following map-level accuracy measures from each of the estimated error matrices, where *C* is the proportion correct, total agreement, or overall accuracy (Eq 10); *D* is the total disagreement (Eq 11); *Q* is the overall quantity disagreement (Eq 12); and *A* is the overall allocation disagreement (Eq 13) [54, 56, 57]:

$$C=\sum_{i=1}^{c}p_{ii}$$ (10)

D=1−C=Q+A (11)

$$Q=\frac{\sum_{i=1}^{c}q_{i}}{2}=\frac{\sum_{i=1}^{c}\left(p_{i+}+p_{+i}\right)\phi_{i}}{\sum_{i=1}^{c}\left(p_{i+}+p_{+i}\right)}$$ (12)

$$A=\frac{\sum_{i=1}^{c}a_{i}}{2}=\frac{\sum_{i=1}^{c}\left(p_{i+}+p_{+i}\right)\alpha_{i}}{\sum_{i=1}^{c}\left(p_{i+}+p_{+i}\right)}$$ (13)

## Results

### Visual similarities and differences between existing topsoil texture maps

While there are some similarities among the seven topsoil texture maps concerning the spatial configuration of topsoil texture areas (i.e., the spatial arrangement, sizes, and shapes of topsoil texture map units), the maps show tremendous differences in the spatial composition of topsoil texture areas (i.e., the number and types of topsoil texture associated with map units) (Fig 2). For example, all maps show a distinct northeast-oriented mushroom-shaped area of uniform topsoil texture in the north-central part of the country. However, the topsoil texture associated with this area varies among the maps: mostly clay in DSMW; loamy sand in NAMSOTER; mostly sandy clay loam in SOTERSAF, SoilGrids1km, and SoilGrids250m; and sandy loam in HWSD and WISE30sec.

**Fig 4:**
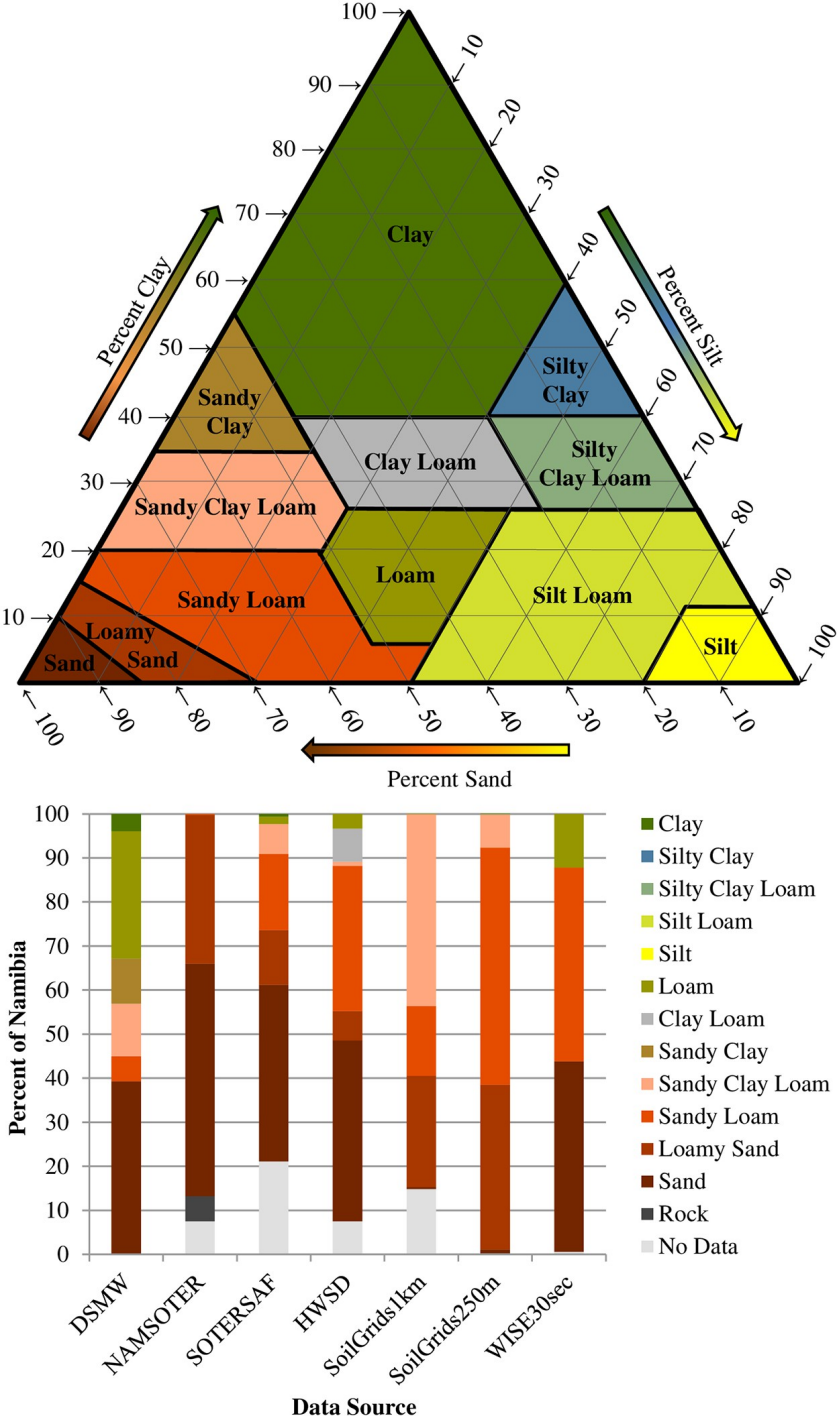
Soil texture triangle (top) and relative areal coverage of dominant topsoil texture types according to the seven map data sources (bottom). The map data sources include DSMW, NAMSOTER, SOTERSAF, HWSD, SoilGrids1km, SoilGrids250m, and WISE30sec. See the text for full names and versions of the map data sources.

The number, types, and spatial extents of topsoil textures vary enormously among the seven maps (Figs 3 and 4). NAMSOTER visibly shows two topsoil texture categories; SoilGrids1km, Soil Grids250m, and WISE30sec three; SOTERSAF five; and DSMW and HWSD six; several of these products include additional topsoil texture classes, but these are too limited in geographic extent to be visible in the figures. Sand makes up around 40% of the map area in DSMW, NAMSOTER, SOTERSAF, HWSD, and WISE30sec, mostly in the eastern part of the country, but only occurs in small pockets in SoilGrids250m. Sandy loam occupies between roughly 16% and 54% of different regions in SOTERSAF, HWSD, SoilGrids1km, SoilGrids250m, and WISE30sec. Loamy sand characterizes anywhere from approximately 12% to 37% in NAMSOTER, SOTERSAF, SoilGrids1km, and SoilGrids250m. Sandy clay loam is common in DSMW, SoilGrids1km, SoilGrids250m, and SOTERSAF, covering about 7% to 44% of diverse portions of different maps. Loam, sandy clay, and clay make up around 42% of the map area in DSWM, but on average less than 2% in all other maps.

The relative prevalence of topsoil textures according to the maps generally reflects that observed in the field, but the maps do not capture the full range of field topsoil textures and several maps have no information on this soil property in some regions (Fig 4, Table 1). The field data show that sand was the most frequent topsoil texture (~ 40%), followed by loamy sand and sandy loam (~ 20% each), and sandy clay loam (~ 10%). Similarly, on average, sand, loamy sand, sandy loam, and sandy clay loam make up about 30%, 15%, 25%, and 10% of the maps, respectively. The field data and all maps also typically agree that clay, loam, clay loam, and sandy clay occur in Namibia but to a limited extent (~ 10% total), and that silty clay, silty clay loam, and silt do not occur at all or perhaps only in small pockets of land. However, the field sampling yielded a few topsoil samples of silt loam, a soil texture that was not represented in any of the maps. In addition, four of the map products (NAMSOTER, SOTERSAF, HWSD, and SoilGrids1km) have between 10% and 20% areas without any topsoil texture information, mostly in the Skeleton Coast, Tsau//Kheib, and Etosha National Parks, where access restrictions have hampered field soil sampling.

Scale of digital maps is a challenging concept [58], but the level of spatial detail clearly varies among the maps and none of the maps comes close to representing the variability of topsoil textures observed in the field (Fig 3). In the vector maps, spatial detail is a function of the number and smallest size (i.e., minimum mapping unit—MMU) of topsoil texture polygons. Ignoring small polygons along Namibia’s border resulting from the spatial subset of data (e.g., Namibia DSMW data were extracted from the global DSMW dataset) and considering only unique topsoil texture polygons (i.e., original mapping units dissolved based on topsoil texture), DSMW has around 33 polygons and an MMU of 818 km^2^, NAMSOTER about 153 polygons and an MMU of 19 km^2^, and SOTERSAF roughly 172 polygons and an MMU of 18 km^2^. In raster maps, spatial detail is typically reported in terms of spatial resolution, which in turn usually corresponds to pixel size. However, two of the raster maps were produced by rasterization of vector maps (i.e., conversion of a vector layer into a raster layer / here disaggregation of polygons into pixels) and so pixel size is a poor measure of the spatial detail in these products. Following vectorization of these two maps (i.e., conversion of a raster layer into a vector layer / here aggregation of neighboring pixels of the same class into polygons) and ignoring problematic small polygons like those noted above, HWSD has around 190 polygons and an MMU of 16 km^2^ and WISE30sec has approximately 119 polygons and an MMU of 11 km^2^. Finally, because each pixel was modeled uniquely in the SoilGrids products, one may argue that each raster pixel is equivalent to a vector polygon, in which case SoilGrids1km has 885,982 pixels (including ~ 15% unclassified pixels) and an MMU of 1 km (spatial resolution ~ 1 km) while SoilGrids250m has 16,610,922 pixels (including ~ 0.21% unclassified pixels) and an MMU of 0.0625 km^2^ (spatial resolution ~ 250 m). These numbers are a bit misleading, however, and difficult to compare to the numbers reported for the other maps. For comparison purposes, vectorized versions of SoilGrids1km and SoilGrids250m have 12,235 and 259,527 polygons, respectively, at the original MMUs of 1 km^2^ and 0.0625 km^2^, respectively.

### Accuracy of existing topsoil texture maps

**Fig 5:**
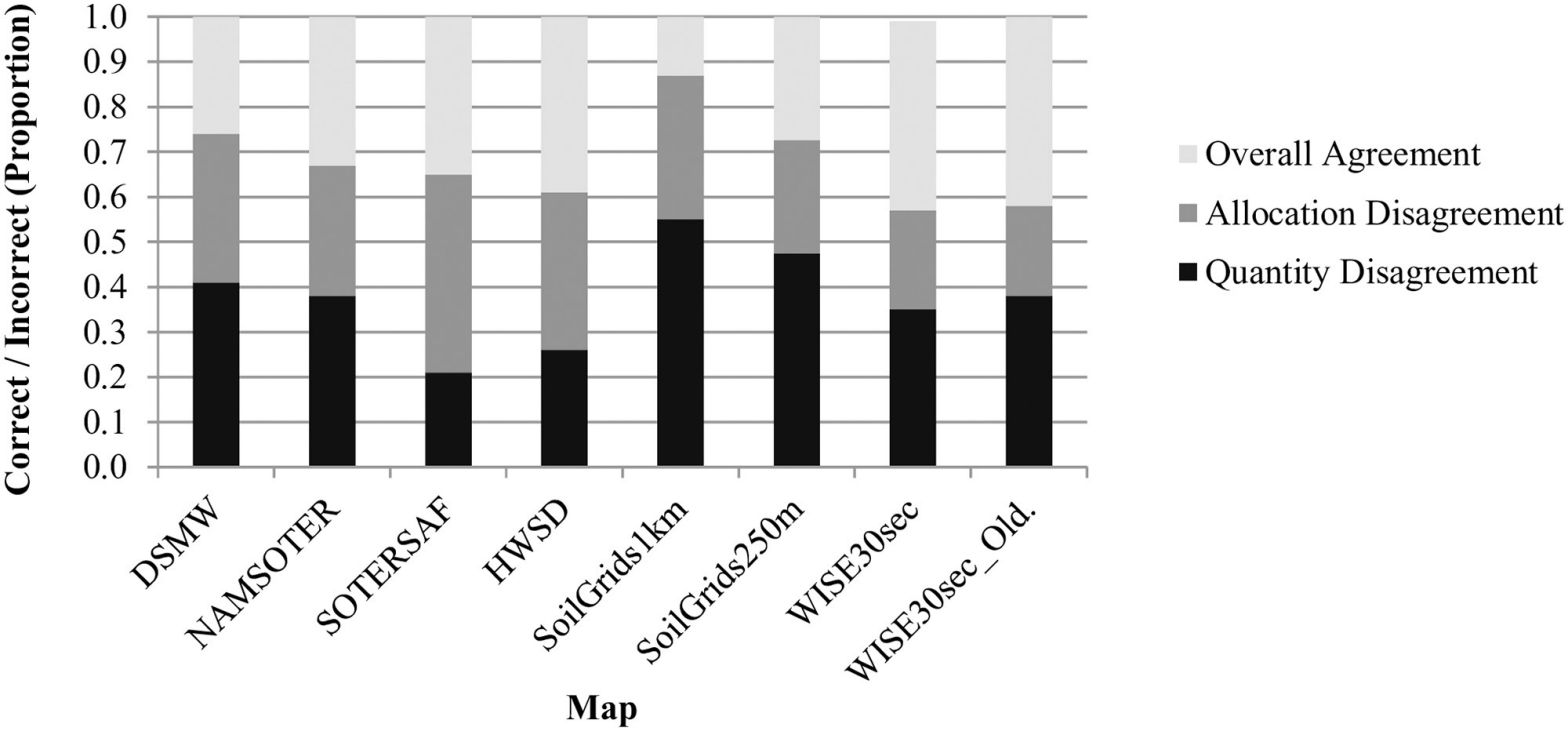
Overall agreement and disagreement between map and field data. See the section on “Topsoil Texture Map Accuracy Assessment” for a definition of overall agreement and disagreement. See the text for full names and versions of the map data sources.

Disagreement between the maps and the field reference data is substantial and generally decreases from the earlier published maps to the more recently published maps (Fig 5). Total disagreement among the map and field data ranges from 58% to 87%. When considering only those maps produced through the linking of digitized map units with soil attribute data, disagreement decreases from the older to the younger maps: from 74% in DSMW, 67% in NAMSOTER, 65% in SOTERSAF, and 61% in HWSD, to 57% in WISE30sec. Similarly, when considering the maps produced through digital soil mapping only, disagreement decreases over time: from 87% in SoilGrids1km to 73% in SoilGrids250m.

**Fig 6:**
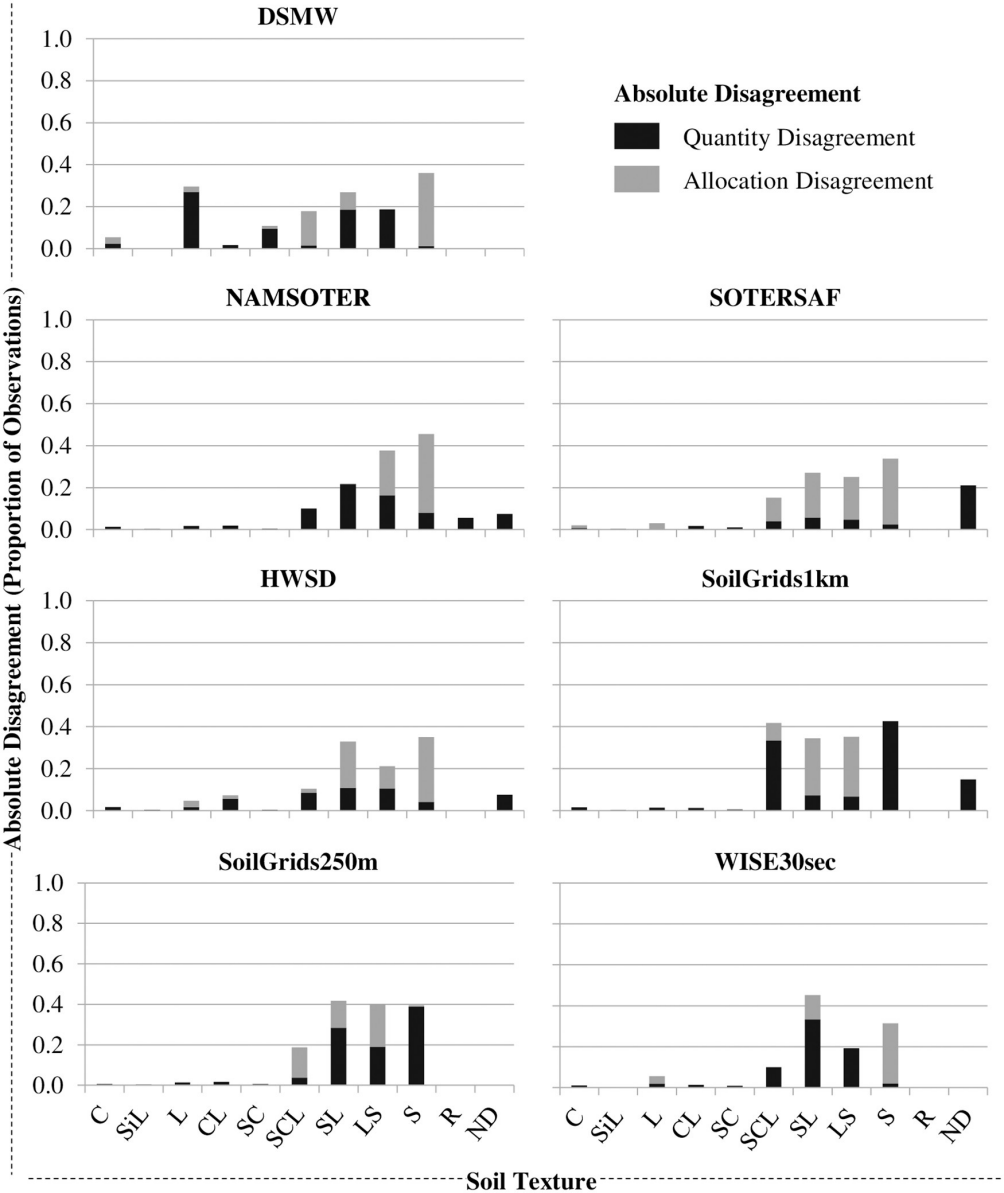
Absolute disagreement between map and field data at the topsoil texture category level. See the section on “Topsoil Texture Map Accuracy Assessment” for a definition of absolute disagreement. SiC, SiCL, and Si are not shown because these were not represented in any of the field samples or maps. See the text for full names and versions of the map data sources.

**Fig 7:**
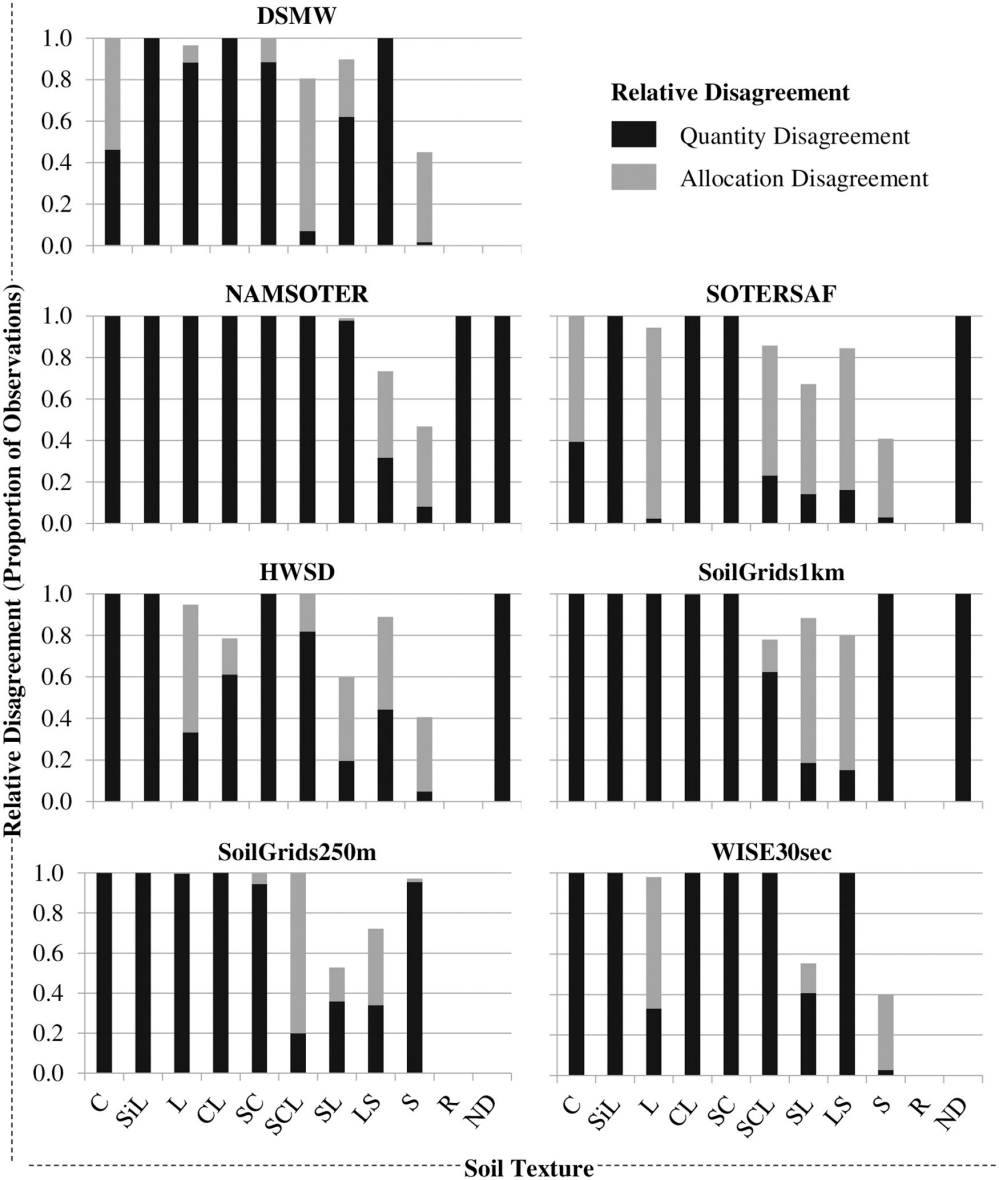
Relative disagreement between map and field data at the topsoil texture category level. See the section on “Topsoil Texture Map Accuracy Assessment” for a definition of relative disagreement. SiC, SiCL, and Si are not shown because these were not represented in any of the field samples or maps. Cases with complete relative quantity disagreement are those in which a soil texture was present in the field data and not the map data (i.e., C, SiL, L, CL, SCL, SL, LS, or S) or in which the map data included categories not present in the field data (i.e., R or ND). See the text for full names and versions of the map data sources.

**Fig 8:**
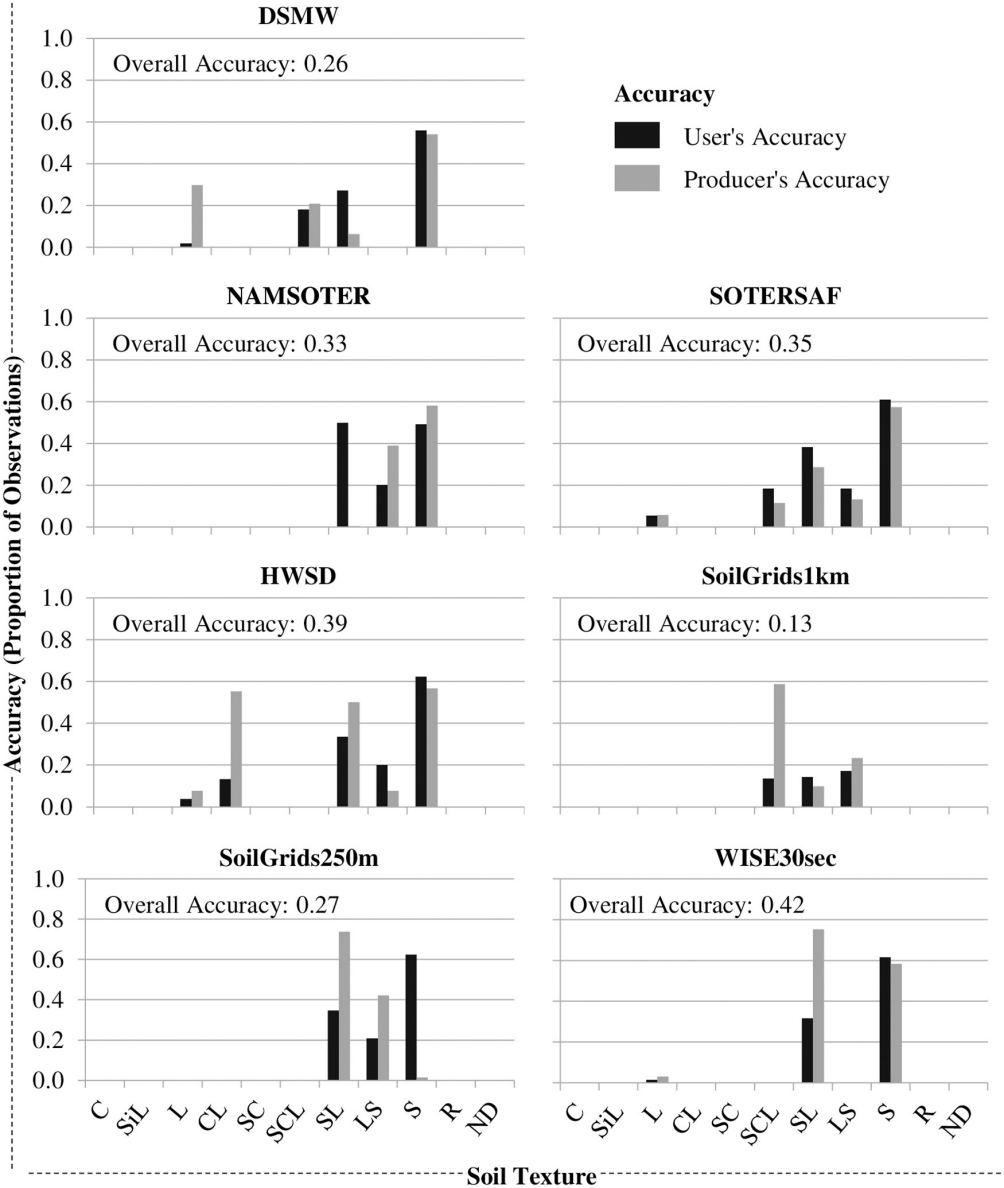
User’s and producer’s accuracies of the different topsoil texture categories in the maps and overall map accuracies. See the section on “Topsoil Texture Map Accuracy Assessment” for definitions of user’s and producer’s accuracies. SiC, SiCL, and Si are not shown because these were not represented in any of the field samples or maps. See the text for full names and versions of the map data sources.

Disagreement among map and field data is due to both less than ideal matches in the proportion and spatial distribution of the topsoil texture categories (Figs 6 and 7), and user’s and producer’s accuracies [Eqs 2 (*υ*_*i*_) and 3 (*π*_*i*_), respectively] are low for most of these categories (Fig 8). In terms of absolute disagreement [Eq 6 (*d*_*i*_)], the contribution of quantity and allocation disagreement [Eqs 4 (*q*_*i*_) and 5 (*a*_*i*_), respectively] to general disagreement is similar (55% and 45%, respectively) (Fig 6). Absolute disagreement generally increases with the areal extent of topsoil texture categories within the map, however, so that coarser topsoil textures generally have higher levels of absolute disagreement (e.g., ~ 40% for sand) than finer topsoil textures. In terms of relative disagreement [Eq 9 (*δ*_*i*_)], the contribution of quantity disagreement to general disagreement is much larger than that of allocation disagreement [79% vs. 21%, respectively; Eqs 7 (*ϕ*_*i*_) and 8 (*α*_*i*_), respectively] (Fig 7). Relative quantity disagreement is 100% for the finer topsoil texture categories in most of the maps, simply because most maps do not account for these categories. In categories represented in the maps, relative disagreement exceeds 40% in all cases and is usually more due to allocation than quantity errors. Given errors in both the mapped proportions and locations of topsoil texture categories, user’s and producer’s accuracies of most categories are low, ranging from 0% in the finer topsoil texture categories to around 60% or 70% in at least one coarser topsoil texture category in each of the maps (Fig 8). There is no clear pattern in user’s and producer’s accuracies with respect to map type or age.

**Fig 9:**
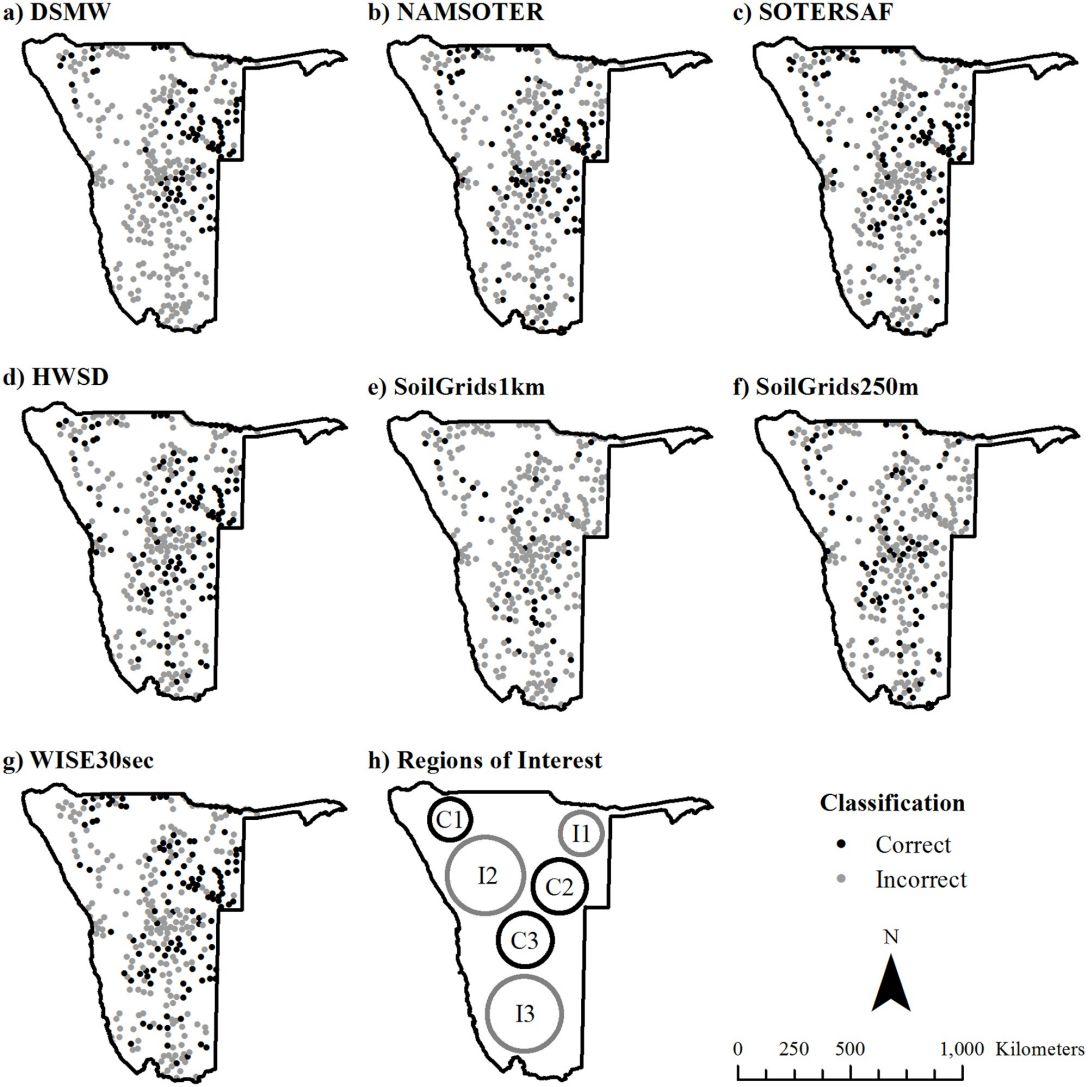
Spatial distribution of correctly and incorrectly classified locations. Panels a-g) show correctly and incorrectly classified locations in: a) DSMW, b) NAMSOTER, c) SOTERSAF, d) HWSD, e) SoilGrids1km, f) SoilGrids250m, and g) WISE30sec. Panel h) shows regions in which locations are often classified correctly (C1, C2, and C3) or incorrectly (I1, I2, and I3). See the text for full names and versions of the map data sources.

Topsoil texture map accuracy varies across space (Fig 9). Most maps show the correct topsoil texture in at least a few locations in all parts of Namibia. However, some regions tend to be classified more accurately than others. In the conventional maps (Fig 9a–9d and 9g), there are a number of accurately mapped sites in the Kunene, Omaheke, and Khomas regions of Namibia (C1, C2, and C3, respectively, in Fig 9h) and a number of inaccurately mapped locations in the Kavango region and much of the northwest-central and southern portions of the country (I1, I2, and I3, respectively, in Fig 9h). In the digital maps (Fig 9e and 9f), there appears to be less of a spatial pattern, though the central part of Namibia has a few more accurately mapped sites than the rest of the country.

The amount and type of confusion between topsoil texture categories differ notably and not systematically among maps and topsoil texture categories, and confusion occurs among all classes (see S2 Text for the error matrices). For example, looking at the data from the map user’s perspective, 40.1% of the SOTERSAF map is classified as sand, but nearly half of this is loamy sand, sandy loam, sandy clay loam, clay loam, silt loam, and clay in the field. Similarly, in Wise30sec, 57.5% of the map is classified as sandy loam, but more than two-thirds of this is sand, loamy sand, sandy clay loam, sandy clay, clay loam, loam, or clay in the field. Alternatively, looking at the data from the map producer’s perspective, the topsoil texture in 22% of all field locations is sandy loam, but 99% of these locations are classified as loamy sand, sand, and rock in NAMSOTER. Similarly, of the 42.6% of all field locations found to have a sandy topsoil, 85% are mapped as sandy clay loam, sandy loam, and loamy sand and 15% are not mapped at all in SoilGrids1km. More examples of category confusion could be given, but the point is that confusion is considerable among both texturally similar and dissimilar topsoils.

## Discussion

### Quality of existing topsoil texture maps

The seven maps agree in some ways but disagree in many more, implying that decisions—and their consequences—will vary tremendously depending upon the map chosen as input. The maps share some similarities in the spatial configuration of map units (Fig 3), which can most likely be attributed to the fact that the more recent maps often used as input the map units of the older maps (Fig 2). The maps also tend to agree with each other and with the field data that Namibia is characterized mostly by coarse-textured topsoils (sand, loamy sand, sandy loam, sandy clay loam) and, to a limited extent, by medium-textured (sandy clay, clay loam, and loam) and fine-textured (silt loam and clay) topsoils (Figs 3 and 4, Table 2). However, the maps disagree substantially with respect to the spatial composition of map units (Figs 3 and 4). That is, there are differences with respect to the number, types, and exact spatial extents of topsoil texture categories, most likely due to the use of different soil profile data, covariate data, and map production methods (Fig 2). As a result, despite certain similarities, the specific topsoil texture observed in any given location differs significantly among the seven maps. Using the various maps as input for decisions may thus produce vastly different outcomes. For example, the mechanical or chemical removal of shrubs from fine-textured soils may result in the restoration of grasses while that from coarse-textured soils may result in the reestablishment of shrubs rather than grasses [59]. Similarly, because soil texture influences soil moisture content, different data on soil texture in a particular location will result in different decisions about when and how much to irrigate and hence crop yields [60].

All of the maps are highly inaccurate, suggesting that their use will likely introduce significant errors into any models or decisions that use them as input. Overall accuracies of the maps range from 13% to 42%, which means that more than half of all locations in each of the maps show the wrong topsoil texture category (Fig 5). Relative quantity disagreement is 100% for most of the finer topsoil texture classes in most of the maps largely because most of the maps simply do not account for these classes at all. Absolute and relative disagreement for the coarser topsoil texture classes in most of the maps is around 40% and 80%, respectively, and mostly due to allocation errors (Figs 6 and 7). User’s and producer’s accuracies are 0% for most categories in most maps and around 60% for the most common coarser topsoil textures in most maps (Fig 8). Confusion in all maps is substantial among all classes, not just classes that are adjacent in the soil texture triangle (Fig 4; S2 Text), suggesting that the maps are often not just somewhat wrong but very wrong. About two-thirds of the maps have no topsoil texture information for 10% to 20% of Namibia (Figs 2 and 4). Clearly, any models or decisions based on the seven maps examined here will be fraught with errors: they may get it right 13% to 42% of the time depending on the map used but, the rest of the time, they may get it wrong, often considerably so.

The maps generally improve over time in both accuracy and spatial detail, but they nonetheless do not capture the variability of topsoil textures observed on the ground, especially in some parts of Namibia. Accuracy increases over time in the conventional maps from 26% to 42% and in the digital maps from 13% to 27% (Fig 5), most likely due to the growth of soil profile databases and the refinement of soil mapping methods. Errors persist, however, at least in part due to the propagation of geometric errors caused by the recycling of older map unit boundaries in the progressively younger maps (Fig 3). With the exception of WISE30sec, spatial detail in the maps also increases over time, from around 33 map units with an MMU of 818 km^2^ in DSMW to around 259,527 map units with an MMU of 0.0625 km^2^ in SoilGrids250m. Still, considering the variability of topsoil textures according to the field data (Fig 3a and 3b), it is evident that the map units are either too generalized to capture the fine-scale variations in topsoil textures (Fig 3c–3g and 3i) and/or that the range of soil textures considered is simply too small (Fig 3g and 3h). Some parts of Namibia are mapped less accurately than others (Fig 9), possibly due to less or poorer field reference data for map production, less homogeneity in topsoil texture types, or prevalence of topsoil textures that are less typical for Namibia.

There are various potential reasons for the observed inaccuracies in the conventional and digital maps discussed above. At the most fundamental level, the conventional maps are extreme generalizations of a much more complex reality. With map scales as low as 1:5,000,000, these maps are incapable of capturing the fine-scale variations in topsoil texture encountered in the field. Most of the conventional maps try to capture such variations by including data on component soils in their associated attribute databases. However, the locations of component soils within map units are usually unknown and so, in reality, are often ignored in efforts using the maps. If we had compared the field data with topsoil textures of the component soils, we might have shown agreements in some locations, potentially increasing map accuracy. Errors in the conventional maps are also likely due to logical inconsistencies resulting from the compilation of multiple maps created by different people at different times at various spatial scales as well as uncertainties associated with the rules and data used to estimate soil properties of map units [2, 5]. With respect to the digital maps, a lack of soil profile data in some regions of the world and/or the low density or spatial clustering of such data is a major hurdle to calibrating more accurate models of soil properties [38]. Another factor contributing to the comparatively high levels of disagreement among field and digital map data is that data of explanatory variables are either unavailable or available at a spatial scale that is much coarser than that at which soil variations occur in the field [38]. Finally, a potential reason for observed errors in both conventional and digital maps is a disagreement regarding what constitutes topsoil (e.g., 0 to 20 cm vs. 0 to 30 cm), which makes the comparison of the datasets not always straightforward.

### Mobile apps, citizen science, and crowdsourcing for improving on-site soil texture estimates

On-site evaluation of soil texture is critical given the notable errors in existing topsoil texture maps. Fortunately, soil texture is an important soil property that can be estimated well, quickly, and inexpensively in the field using the “texture-by-feel method” [61]. In this method, the user takes a soil sample of about 25 g and estimates texture by rolling, squeezing, and rubbing the soil between his or her fingers. The texture-by-feel method can be nearly as accurate as laboratory analyses when completed by trained personnel and is also less expensive and time-consuming [17]. Even when used by less experienced personnel, users have found it to be sufficiently accurate for a diversity of purposes. Several studies have found that users can estimate the correct soil texture class using this method about half of the time [62–65]. While this level of agreement is not high, it is not a major issue for many applications, because the estimates differ by just one texture class, a difference that would occur randomly at least some of the time anyway, due to lab measurement errors for textures near class boundaries. In a recent study involving thirty-seven different soil texture classes, for example, estimators were able to place 87% of all samples in the correct or adjacent soil texture class [17]. This value can be expected to be even higher when only twelve USDA soil texture classes are used. Moreover, errors in field estimates in earlier studies were often associated with issues such as high amounts of coarse fragments and organic matter, both of which can be minimized through careful sample preparation in the field. Clearly, given the low accuracy of existing topsoil texture maps, the texture-by-feel method provides a viable cost-effective, time-efficient, and for most purposes sufficiently accurate alternative to laboratory analyses.

Mobile apps can be used to both facilitate and improve collection of soil texture and other data in the field [66–70]. Through their collection of volunteered geographic information, the apps facilitate crowdsourcing for geographic knowledge production—a powerful tool as is illustrated by LandPKS [66, 71], a citizen science and crowdsourcing effort aimed at estimating land potential based on site-level soil and vegetation cover characterization. The LandPKS app has characterized over 20,000 soil profiles globally, with the number of sites rapidly increasing as its user base grows. App-based data collection is facilitated by providing standard data entry fields and choice lists, and by automatically submitting the data to cloud-based data storage, curation, and retrieval systems. Data collection can be improved by integrating decision support tools into the apps, including flowcharts and embedded training videos. For example, the LandPKS app includes a LandInfo module [66, 71] that guides the user through the process of determining soil texture using the texture by feel method with a series of questions illustrated by simple animated videos. Once the field data collection is complete, users may download the data in spreadsheet format from the data portal on the LandPotential.org website, where the data may be edited and an API is also available.

Crowdsourced soil data can be integrated into digital or conventional soil mapping workflows, where volunteered data that has met some minimum Quality Assurance / Quality Control (QA/QC) standard can be used to supplement existing soil point data for creating or updating soil maps. A recent example of this is the iSDAsoil map of Africa [72] which used approximately 12,000 LandPKS soil profile observations in their prediction of soil particle size classes (i.e., sand, silt, clay). Crowdsourced soil data are often collected as generalized class-based soil property estimates (e.g., soil texture class) which lack the resolution of quantitative soil property data commonly used in soil maps. In their incorporation of LandPKS soil data, the iSDAsoil project overcame this issue by converting texture classes to soil particle size fractions using the particle class values at the soil texture class centroids. Novel strategies like this allow for the incorporation of crowdsourced soil data with traditional soil data sets and have the potential to significantly improve soil map accuracy.

### Improving soil map predictions

Increasing rates of soil degradation across the globe have highlighted the need for accurate soil information that can inform site-specific soil management [73]. Results from this study clearly demonstrate that existing soil maps lack the required accuracy needed to direct sustainable land management at the farm-scale. While improvements in mapping technologies have resulted in more accurate soil maps, as shown above for both digital and conventional soil maps (Fig 5), there are still areas where significant improvements can be made. These areas include (i) improvement of soil models, (ii) increasing the number of soil observations and their representation of soil variability, and (iii) improving the spatial resolution and types of environmental covariates used to model soil spatial variability.

Recent advancements in soil modeling have occurred for both digital and conventional soil mapping. For digital soil mapping, advances in machine learning, and in particular ensemble modeling [74–76], have resulted in improvements in model accuracy. Recent digital soil models also provide spatial estimates of model uncertainty [72, 77], which can help end-users assess how reliable a model might be in a certain area. Likewise, recent improvements in conventional soil mapping are leading to improved spatial accuracy. Conventional soil maps are often limited in their usefulness due to the spatial ambiguity of individual soil components within soil map unit polygons and the semantic description of soil information within the soil map database. However, recent advancements in soil map unit disaggregation (i.e., delineation of soil components within map unit polygons) and soil data mining now provide methods for translating expert soil knowledge into usable soil information [78–80]. For example, several recent studies have leveraged the soil-landscape relationships recorded in soil surveys to distinguish component soils within map units [80, 81] while others have developed automated methods for soil map unit disaggregation [82, 83]. These efforts are producing spatially refined conventional soil maps that can be used either directly or as input data for new digital soil maps.

Additional advances are necessary in the collection of data to produce soil maps that can be used reliably in local to global applications (e.g., farm-level land management to global climate change modeling). One area of concern in this context is sampling. As Brevik et al. [2] point out, “increases in mapping efficiency […] should not be mistaken for a reason to invest fewer resources to fieldwork.” In fact, additional field data are critical for enhancing our ability to calibrate and evaluate soil predictions. As a first step, these data may be collected in areas that are currently unsampled or undersampled or in which mapping errors are common (Fig 9). Eventually, to optimize model calibration, field data should capture the heterogeneity of factors influencing soil formation in the study region of interest. The sampling approach itself should also be flexible and efficient [25]. In addition to these carefully sampled soil data sets, crowdsourced data can play a crucial role in increasing the number of soil observations and their representation of soil variability, as described in the previous section. Many mobile apps like *LandPKS* above are free, simple, and effective instruments with tremendous potential for supporting both traditional and crowdsourced soil sampling efforts.

In addition to sampling, we also need to identify new or better and higher spatial resolution covariates for digital and conventional soil maps. Ideally, these data layers should “account for anthropogenic and natural forcings that determine and modulate soils” [5] and be “complete, consistent, and as correct and current as possible” [38]. Even with more and better soil and covariate data in hand, however, it will still be challenging to produce predictions that meet the needs of all stakeholders in soil resources research and management.

One way forward, in addition to the development of enhanced soil models, better soil observations, and improved covariates, will be the rigorous and comprehensive comparison of new methodological approaches [e.g., 84–86]. That is, we need to assess the strengths and limitations of different approaches with respect to their underlying assumptions, computational requirements, robustness, prediction accuracies, and uncertainties. Soil map quality assessment, another important research topic, will play a critical role in this endeavor. Other key research topics include spatial decomposition and/or lagging of soil and covariate data, (re)presentation of digital soil maps, and economics of digital soil mapping [87] as well as optimization of computing efficiencies [44]. Finally, Grundwald, Thompson, and Boettinger [5] argue that we need to identify an ideal “soil pixel”, i.e., a pixel that provides detailed information about multiple soil properties at appropriate spatial and temporal resolutions in a contiguous fashion across space and time.

To improve soil map predictions, we also need to fully capitalize on trends and developments that are happening in disciplines affiliated with soil science [2, 7, 25, 88, 89]. Continued advances in geographic information science and technology offer new tools and techniques for soil data mining, analysis, and modeling on a regular basis. The development of proximal sensing tools and technologies for measuring soil properties in the field (e.g., ground-penetrating radar and optical sensors) also promotes the quantitative characterization of soils [90, 91]. Moreover, digital spatial data such as high-spatial resolution climatologies [e.g., 92, 93] are becoming increasingly available to support pedometrics, often at no cost. Advances in computational capabilities, including both desktop and cloud computing, facilitate the processing of increasingly large datasets using increasingly sophisticated algorithms. Finally, as described above, developments in citizen science, mobile technologies, and online services offer new opportunities for digital soil mapping, conventional soil mapping, or hybrid approaches.

## Conclusions

Accurate high spatial resolution soil information is urgently needed by a diversity of stakeholders to address issues ranging from local to global scales (e.g., farm-level land management to global climate change adaptation and mitigation). However, the quality of existing soil maps is largely unknown. To address this problem, we evaluated the quality of seven spatial soil databases (DSMW, NAMSOTER, SOTERSAF, HWSD, SoilGrids1km, SoilGrids250m, and WISE30sec) using topsoil texture as an example soil property and Namibia as a case study area. We found that the maps ranged in overall accuracies from only 13% to 42%, with substantial confusion occurring among all texture categories, not just those in close proximity in the soil texture triangle. Visual comparisons of the maps moreover showed that the maps differ greatly in the spatial composition and configuration of topsoil texture areas.

The use of these existing maps for policy- and decision-making is thus highly questionable. More specifically, we draw three major conclusions. First, existing soil maps are often insufficient to support local land management and problematic for the use in global models of climate change, biodiversity, and ecosystem services. Any use of soil maps should consequently be preceded by their careful evaluation for an intended purpose. Second, to address many of today’s issues in a sensible manner, we need soil data that are up-to-date, sufficiently accurate for their intended purpose, associated with uncertainty information, three-dimensional, available at multiple spatial resolutions, spatio-temporally explicit and continuous, affordable, easily integrated with other digital spatial data, and readily available for interested stakeholders. Third, to generate soil data that meet the requirements of the diverse stakeholders in soil resources research and management, we need to improve on-site estimates and map predictions of soil properties.

We suggest that mobile apps, citizen science, and crowdsourcing can help meet this need. Better on-site estimates of soil properties may be obtained through use of free and open source mobile and web apps that facilitate the collection and recording of georeferenced data on various soil properties and other site characteristics. Better soil map predictions may be generated by taking greater advantage of crowdsourced soil data and implementing a range of strategies to improve model performance (e.g., collect more field data, find new and better covariates, develop novel quantitative models for making spatial soil predictions, enhance uncertainty and error assessments, and optimize computing efficiencies).

## Supporting information

S1 Text Description of soil databases. This document contains additional information on each of the seven soil databases considered in this study.
(DOCX)

S2 Text Error matrices. This document contains the error matrices from the accuracy assessment.
(DOCX)

S1 Dataset Field soil data. This document contains geographic coordinates and soil texture information for the 327 field samples used for assessing the accuracy of the soil texture maps presented in this manuscript.
(XLSX)
