# Supplementary material for: Errors in soil maps: The need for better on-site estimates and soil map predictions
Source: PLoS One. 2023 Jan 11;18(1):e0270176. doi: 10.1371/journal.pone.0270176 (PMC9833593; doi:10.1371/journal.pone.0270176)
Supplement: S1 Text — This document contains additional information on each of the seven soil databases considered in this study. (DOCX) [file pone.0270176.s001.docx]

## Description of Soil Databases

The DSMW (Version 3.6) is a vector dataset of soils across the globe at a map scale of 1:5,000,000 (1). Produced by the FAO, it represents a digital version of the printed FAO-UNESCO Soil Map of the World (2). This original map was published between 1974 and 1978 at a map scale of 1:5,000,000 and represents the outcome of an enormous data integration effort, including the compilation and harmonization of some six hundred multi-scale and multi-legend soil maps through field and lab work and tremendous expert input. The DSMW is comprised of 106 soil units (3), each of which includes a dominant soil and many of which include up to seven additional component soils. That is, many of the soil units in this map are not homogenous and include associated soils (soils making up more than 20% of map unit) and inclusions (soils making up less than 20% of map unit) in addition to the dominant soil. The DSMW contains a database of numerous soil attributes for the topsoil (0-30 cm) and subsoil (30-100 cm) of each its 4,931 mapping units, including information on soil texture of the dominant soil. Quantitative information on DSMW accuracies or uncertainties is currently unavailable.

NAMSOTER and SOTERSAF, the next two map products in our collection, were developed as part of the World SOil and TERrain (SOTER) Digital Database Programme (4, 5). Initiated in 1986, SOTER is implemented by FAO, ISRIC, and the United Nations Environment Programme (UNEP); sponsored by the International Union of Soil Sciences (IUSS); produced at a map scale of 1:5,000,000; and intended to replace the DSMW upon global coverage. SOTER methodology involves the digitization and coding of map units called SOTER units, the creation of a soil and terrain attribute database, and the linkage of these map and tabular data. SOTER units are delineated in a hierarchical fashion using geospatial data such as digital elevation models and satellite imagery, beginning with landforms at the most general level, then parent material, then terrain components, and finally soil components at the most detailed level. Soil and terrain attributes are coded according to the SOTER Procedures Manual (5). Initial information about soil attributes comes from typical (real) soil profiles. In the case of missing data, gaps in the primary SOTER databases are filled using additional soil data, expert knowledge, and a taxotransfer rule-based procedure that draws on soil physical and chemical data held in the ISRIC-WISE Global Soil Profile Database (6-8). This process results in a separate secondary dataset, the SOTWIS database (9). SOTER/SOTWIS soils are described at standard depths of 20 cm intervals from 0 to 100 cm depth in terms of various characteristics, including USDA texture classes. If soil and terrain components occur at scales too fine for the desired mapping scale, the dominant components are stored in both the geometric and attribute databases while all non-dominant components are stored in the attribute database only. Quantitative information on the accuracies or uncertainties of SOTER-related map products is currently unavailable.

NAMSOTER (Version 1.0) is a vector dataset and attribute soil and terrain database of Namibia, produced by the AEZ Programme of the MAWRD as part of Namibia’s National Soil Survey (10, 11). NAMSOTER is the Namibian version of SOTER and includes a national dataset at a map scale of 1:1,000,000 (used here) as well as finer-scale datasets of Growing Period Zones 2 and 3 in the northeastern part of the country (1:250,000); the Okavango River Terrace in the northern Kavango region (1:100,000); and the northeastern Omusati, western Ohangwena, northern Oshana, and northwestern Oshikoto regions (1:100,000) (11). Three areas of Namibia are not currently mapped in NAMSOTER, mostly due to access restrictions, including the Skeleton Coast National Park, the Tsau//Kheib National Park (former Sperrgebiet), and Etosha National Park. NAMSOTER includes 92 SOTER units composed of 118 terrain components and 269 soil components as well as 56 representative georeferenced soil profiles classified according to the Revised Legend (12) and the World Reference Base (13, 14). Of the 321 mapping units, most include at least one and some up to six associated soils or soil inclusions in addition to the dominant soil.

SOTERSAF (Version 1.0) is a vector dataset and attribute soil and terrain database of eight southern African countries: Angola, Botswana, Mozambique, Namibia, Tanzania, South Africa, Swaziland, and Zimbabwe (15-17). The outcome of collaboration among national soil institutes in southern Africa, individual soil experts, FAO, ISRIC, and UNEP, SOTERSAF was produced at a map scale of 1:2,000,000 through the harmonization of existing national SOTER datasets. SOTERSAF includes 4,019 SOTER units composed of 8,445 terrain components and 15,076 soil components as well as 941 representative georeferenced soil profiles classified according to the Revised Legend (12) and the World Reference Base for Soil Resources (13, 14). Of the 6,099 mapping units, most include at least one and some up to six associated soils or soil inclusions in addition to the dominant soil.

The HWSD (Version 1.21) is a raster dataset of soils across the globe at a spatial resolution of 30 × 30 arc-seconds (18, 19). It is the result of a collaborative effort between the FAO, the International Institute for Applied Systems Analysis (IIASA), ISRIC, the European Soil Bureau Network of the Joint Research Centre of the European Commission (ESBN-JRC), and the Institute of Soil Science of the Chinese Academy of Sciences (ISS-CAS). The HWSD was produced by integrating the soil map units of four major national and international map products (46% DSMW, 24% SOTER, 24% European Soil Database, 6% Soil Map of China) with soil property estimates obtained through the standardized application of expert knowledge and taxonomy-based taxotransfer rules to soil profiles from the ISRIC-WISE Global Soil Profile Database (8) and other sources. The HWSD encompasses 16,382 soil map units, each containing up to nine soil map unit / topsoil texture combination records and information on soil parameters for the topsoil (0-30 cm) and subsoil (30-100 cm), including USDA soil textural classes and sand, silt, and clay fractions. Quantitative information on HWSD accuracies or uncertainties is currently unavailable.

SoilGrids1km is a raster world soils dataset at a spatial resolution of 1 × 1 km (20, 21). It is a component of ISRIC’s Global Soil Information Facilities (GSIF) cyberinfrastructure, was produced through automated mapping, and provides estimates of ten major soil properties (e.g., sand, silt, and clay content) and two major groups of soil classes (WRB soil groups and USDA Soil Taxonomy suborders) at six standard depths (0-5 cm, 5-15 cm, 15-30 cm, 30-60 cm, 60-100 cm, 100-200 cm). Different regression and/or regression-kriging approaches were used for predicting different soil properties and classes (e.g., multiple linear regression was used for estimating sand, silt, and clay content). Input data for the mapping included publicly available soil profile data at point observations for model calibration and evaluation as well as spatially explicit and continuous data as explanatory layers. Profiles of roughly 110,000 points from a diversity of national and international soil profile databases (e.g., ISRIC-WISE and SOTER) were used in the production of SoilGrids1km as well as 75 covariates representing soil forming factors and other potential predictors (e.g., HWSD soil map units, elevation, and land cover). The mapping accuracy for the different soil properties was estimated quantitatively using amount of variation explained by the models and varied by soil property (e.g., regression models accounted for 23.5%, 34.9%, and 24.4% of observed variability in sand, silt, and clay content, respectively).

SoilGrids250m is a raster world soils dataset at a spatial resolution of 250 × 250 m (22, 23). The product was generated through automated mapping, and provides estimates of nine major soil properties (e.g., sand, silt, and clay content) and two major groups of soil classes (USDA Soil Taxonomy suborders and WRB soil groups) at seven standard depths (0, 5, 15, 30, 60, 100, and 200 cm). Different machine learning approaches were used for predicting different soil properties and classes (e.g., estimates of sand, silt, and clay content were generated using an ensemble of random forest and gradient boosting methods). As in SoilGrids1km, soil profile data at point locations were used for model calibration and evaluation while a diversity of spatially explicit and continuous data layers served as covariates. The point data came from about 150,000 soil profiles, which were compiled from various national and international soil profile databases and most of which are available via ISRIC’s World Soil Information Service (24). A set of 158 environmental covariates were considered in the model, including various topographic, climatic, and land cover layers. The mapping accuracy for the different soil properties was estimated quantitatively using 10-fold repeated cross-validation and varied by soil property (e.g., the models accounted for 78.6%, 79.4%, and 72.6% for sand, silt, and clay, respectively).

WISE30sec (Version 1.0) is a raster dataset of soils across the globe at a spatial resolution of 30 × 30 arc-seconds (25-27). It was produced at ISRIC by linking combined HWSD soil / Köppen-Geiger climate class units with soil parameter estimates derived from some 21,000 soil profiles in the ISRIC-WISE Global Soil Profile Database (8) using taxotransfer procedures. The soil analytical data in WISE30sec represent estimates for virtual (synthetic) profiles as defined by their soil/climate type and are available at 20 cm depth increments from 0 to 100 cm depth and at 50 cm depth increments from 100 to 200 cm depth. WISE30sec contains data on twenty soil variables for each of the one to ten components of each of the 16,414 map units, including the distribution of sand, silt, and clay particle sizes. Quantitative information on the accuracy of WISE30sec parameter estimates is currently unavailable, but the dataset has information on uncertainties in the form of standard deviations from the mean of parameter estimates.

## References

1. FAO. Digital Soil Map of the World, Version 3.6 2003 [Available from: <http://www.fao.org/geonetwork/srv/en/metadata.show?id=141161>.

2. FAO-UNESCO. FAO-UNESCO Soil Map of the World, 1:5,000,000, Volumes 1 to 10. Paris, France: FAO/UNESCO; 1971-1981. 346 p.

3. FAO-UNESCO. FAO-UNESCO Soil Map of the World, 1:5,000,000, Volume 1, Legend. Paris, France: FAO/UNESCO; 1974. 346 p.

4. van Engelen VWP. SOTER: The World Soils and Terrain Database. In: Sumner ME, editor. Handbook of soil science. Boca Raton, FL: CRC Press; 1999. p. H19-H28.

5. van Engelen VWP, Dijkshoorn JA. Global and National Soils and Terrain Digital Databases (SOTER), Procedures Manual, Version 2.0. Wageningen, The Netherlands: ISRIC; 2013. 198 p.

6. Batjes NH. A taxotransfer rule-based approach for filling gaps in measured soil data in primary SOTER databases, Version 1.1. Wageningen, The Netherlands: ISRIC; 2003.

7. Batjes NH. Harmonized soil profile data for applications at global and continental scales: updates to the WISE database. Soil Use and Management. 2009;25(2):124-7.

8. ISRIC. ISRIC-WISE - Global Soil Profile Data, Version 3.1 2016 [Available from: <http://www.isric.org/data/isric-wise-global-soil-profile-data-ver-31>.

9. ISRIC. Harmonized Continental SOTER-Derived Database (SOTWIS) 2016 [Available from: <http://www.isric.org/projects/harmonized-continental-soter-derived-database-sotwis>.

10. ICC, MAWRD. Project to support the Agro-Ecological Zoning (AEZ) Programme in Namibia. Windhoek, Namibia: MAWRD; 2000. 243 p.

11. Coetzee ME. NAMSOTER: a SOTER database for Namibia. Windhoek, Namibia: MAWRD; 2001.

12. FAO, UNESCO. FAO/UNESCO Soil Map of the World, Revised Legend. Rome, Italy: FAO; 1988.

13. ISSS, ISRIC, FAO. World reference base for soil resources. Rome, Italy: FAO; 1998.

14. IUSS. World reference base for soil resources: international soil classification system for naming soils and creating legends for soil maps. Rome, Italy: FAO; 2015.

15. Dijkshoorn JA. SOTER database for southern Africa (SOTERSAF). Wageningen, The Netherlands: ISRIC; 2003. 32 p.

16. Batjes NH. SOTER-based soil parameter estimates for Southern Africa. Wageningen, The Netherlands: ISRIC - World Soil Information; 2004.

17. ISRIC. Soil and Terrain Database for Southern Africa (SOTERSAF), Version 1.0 2003 [Available from: <http://www.isric.org/data/soil-and-terrain-database-southern-africa-ver-10-sotersaf>.

18. Nachtergaele F, van Velthuize H, Verelst L, Wiberg D. Harmonized World Soil Database, Version 1.2: FAO, IIASA, ISRIC, ISSCAS, and JRC; 2012.

19. FAO, IIASA, ISRIC, ISSCAS, JRC. Harmonized World Soil Database, Version 1.21 2012 [Available from: <http://webarchive.iiasa.ac.at/Research/LUC/External-World-soil-database/HTML/HWSD_Data.html?sb=4>.

20. Hengl T, de Jesus JM, MacMillan RA, Batjes NH, Heuvelink GBM, Ribeiro E, et al. SoilGrids1km: global soil information based on automated mapping. PLOS ONE. 2014;9(8):17.

21. ISRIC. SoilGrids 1km: An Automated System for Global Soil Mapping 2013 [Available from: <http://soilgrids1km.isric.org/>.

22. Hengl T, de Jesus JM, Heuvelink GBM, Gonzalez MR, Kilibarda M, Blagotic A, et al. SoilGrids250m: global gridded soil information based on machine learning. PLOS. Under Review.

23. ISRIC. SoilGrids 250m 2016 [Available from: [www.soilgrids.org](http://www.soilgrids.org).

24. ISRIC. ISRIC World Soil Information Service (WoSIS) 2016 [Available from: <http://www.isric.org/data/wosis>.

25. Batjes NH. World Soil Property Estimates for Broad Scale Modelling (WISE30sec), Version 1.0. Wageningen, The Netherlands: ISRIC; 2015. 52 p.

26. Batjes NH. Harmonized soil property values for broad-scale modelling (WISE30sec) with estimates of global soil carbon stocks. Geoderma. 2016;269:61-8.

27. ISRIC. ISRIC-WISE Derived Soil Properties on a 30 by 30 Arcsec Global Grid (WISE30sec), Version 1.0 2015 [Available from: <http://www.isric.org/data/isric-wise-derived-soil-property-estimates-30-30-arcsec-global-grid-wise30sec>.
