# Supplementary material for: Errors in soil maps: The need for better on-site estimates and soil map predictions
Source: PLoS One. 2023 Jan 11;18(1):e0270176. doi: 10.1371/journal.pone.0270176 (PMC9833593; doi:10.1371/journal.pone.0270176)
Supplement: S2 Text — This document contains the error matrices from the accuracy assessment. (DOCX) [file pone.0270176.s002.docx]

**Error matrices**

Note: numbers in these error matrices do not always add up to 100% because in some cases, maps had areas with a dominant topsoil texture that was not captured by any of the field samples (Areal coverage of texture classes in map is taken into account to compute matrix.). SiC, SiCL, and Si are not shown because none of the field samples had these topsoil textures and none of the maps showed these texture classes in the locations of the field samples.

**DSMW**

|  |  | **Field Data** | | | | | | | | | | |  |
| --- | --- | --- | --- | --- | --- | --- | --- | --- | --- | --- | --- | --- | --- |
|  |  | **C** | **SiL** | **L** | **CL** | **SC** | **SCL** | **SL** | **LS** | **S** | **R** | **ND** | **SUM** |
| **Map Data** | **C** | 0.0 | 0.0 | 1.0 | 0.5 | 0.0 | 0.0 | 1.0 | 1.5 | 0.0 | 0.0 | 0.0 | 3.9 |
|  | **SiL** | 0.0 | 0.0 | 0.0 | 0.0 | 0.0 | 0.0 | 0.0 | 0.0 | 0.0 | 0.0 | 0.0 | 0.0 |
|  | **L** | 0.5 | 0.0 | 0.5 | 0.3 | 0.3 | 5.9 | 9.4 | 5.4 | 6.7 | 0.0 | 0.0 | 28.9 |
|  | **CL** | 0.0 | 0.0 | 0.0 | 0.0 | 0.0 | 0.0 | 0.0 | 0.0 | 0.0 | 0.0 | 0.0 | 0.0 |
|  | **SC** | 0.0 | 0.0 | 0.0 | 0.0 | 0.0 | 0.9 | 2.7 | 0.9 | 5.8 | 0.0 | 0.0 | 10.3 |
|  | **SCL** | 0.4 | 0.0 | 0.0 | 0.7 | 0.4 | 2.2 | 4.0 | 0.7 | 3.6 | 0.0 | 0.0 | 11.9 |
|  | **SL** | 0.0 | 0.0 | 0.0 | 0.0 | 0.0 | 0.0 | 1.6 | 1.6 | 2.6 | 0.0 | 0.0 | 5.7 |
|  | **LS** | 0.0 | 0.0 | 0.0 | 0.0 | 0.0 | 0.0 | 0.0 | 0.0 | 0.0 | 0.0 | 0.0 | 0.0 |
|  | **S** | 0.6 | 0.3 | 0.3 | 0.3 | 0.0 | 1.4 | 5.8 | 8.8 | 22.0 | 0.0 | 0.0 | 39.3 |
|  | **R** | 0.0 | 0.0 | 0.0 | 0.0 | 0.0 | 0.0 | 0.0 | 0.0 | 0.0 | 0.0 | 0.0 | 0.0 |
|  | **ND** | 0.0 | 0.0 | 0.0 | 0.0 | 0.0 | 0.0 | 0.0 | 0.0 | 0.0 | 0.0 | 0.0 | 0.0 |
|  | **SUM** | 1.4 | 0.3 | 1.8 | 1.8 | 0.6 | 10.3 | 24.3 | 18.8 | 40.7 | 0.0 | 0.0 | 100.0 |

**NAMSOTER**

|  |  | **Field Data** | | | | | | | | | | |  |
| --- | --- | --- | --- | --- | --- | --- | --- | --- | --- | --- | --- | --- | --- |
|  |  | **C** | **SiL** | **L** | **CL** | **SC** | **SCL** | **SL** | **LS** | **S** | **R** | **ND** | **SUM** |
| **Map Data** | **C** | 0.0 | 0.0 | 0.0 | 0.0 | 0.0 | 0.0 | 0.0 | 0.0 | 0.0 | 0.0 | 0.0 | 0.0 |
|  | **SiL** | 0.0 | 0.0 | 0.0 | 0.0 | 0.0 | 0.0 | 0.0 | 0.0 | 0.0 | 0.0 | 0.0 | 0.0 |
|  | **L** | 0.0 | 0.0 | 0.0 | 0.0 | 0.0 | 0.0 | 0.0 | 0.0 | 0.0 | 0.0 | 0.0 | 0.0 |
|  | **CL** | 0.0 | 0.0 | 0.0 | 0.0 | 0.0 | 0.0 | 0.0 | 0.0 | 0.0 | 0.0 | 0.0 | 0.0 |
|  | **SC** | 0.0 | 0.0 | 0.0 | 0.0 | 0.0 | 0.0 | 0.0 | 0.0 | 0.0 | 0.0 | 0.0 | 0.0 |
|  | **SCL** | 0.0 | 0.0 | 0.0 | 0.0 | 0.0 | 0.0 | 0.0 | 0.0 | 0.0 | 0.0 | 0.0 | 0.0 |
|  | **SL** | 0.0 | 0.0 | 0.1 | 0.0 | 0.0 | 0.1 | 0.1 | 0.0 | 0.0 | 0.0 | 0.0 | 0.2 |
|  | **LS** | 0.5 | 0.0 | 0.3 | 0.5 | 0.5 | 6.3 | 10.1 | 6.8 | 8.7 | 0.0 | 0.0 | 33.8 |
|  | **S** | 0.9 | 0.3 | 0.6 | 0.6 | 0.0 | 2.9 | 10.9 | 10.7 | 25.9 | 0.0 | 0.0 | 52.7 |
|  | **R** | 0.0 | 0.0 | 0.8 | 0.8 | 0.0 | 0.8 | 0.8 | 0.0 | 2.4 | 0.0 | 0.0 | 5.7 |
|  | **ND** | 0.0 | 0.0 | 0.0 | 0.0 | 0.0 | 0.0 | 0.0 | 0.0 | 7.6 | 0.0 | 0.0 | 7.6 |
|  | **SUM** | 1.4 | 0.3 | 1.7 | 1.9 | 0.5 | 10.0 | 22.0 | 17.5 | 44.6 | 0.0 | 0.0 | 100.0 |

**SOTERSAF**

|  |  | **Field Data** | | | | | | | | | | |  |
| --- | --- | --- | --- | --- | --- | --- | --- | --- | --- | --- | --- | --- | --- |
|  |  | **C** | **SiL** | **L** | **CL** | **SC** | **SCL** | **SL** | **LS** | **S** | **R** | **ND** | **SUM** |
| **Map Data** | **C** | 0.0 | 0.0 | 0.0 | 0.0 | 0.0 | 0.6 | 0.0 | 0.0 | 0.0 | 0.0 | 0.0 | 0.6 |
|  | **SiL** | 0.0 | 0.0 | 0.0 | 0.0 | 0.0 | 0.0 | 0.0 | 0.0 | 0.0 | 0.0 | 0.0 | 0.0 |
|  | **L** | 0.0 | 0.0 | 0.1 | 0.0 | 0.0 | 0.0 | 0.5 | 0.5 | 0.6 | 0.0 | 0.0 | 1.7 |
|  | **CL** | 0.0 | 0.0 | 0.0 | 0.0 | 0.0 | 0.0 | 0.0 | 0.0 | 0.0 | 0.0 | 0.0 | 0.0 |
|  | **SC** | 0.0 | 0.0 | 0.0 | 0.0 | 0.0 | 0.0 | 0.0 | 0.0 | 0.0 | 0.0 | 0.0 | 0.0 |
|  | **SCL** | 0.0 | 0.0 | 0.5 | 0.3 | 0.0 | 1.3 | 2.3 | 1.3 | 1.3 | 0.0 | 0.0 | 6.8 |
|  | **SL** | 0.0 | 0.0 | 0.0 | 0.3 | 0.3 | 3.5 | 6.6 | 4.3 | 2.3 | 0.0 | 0.0 | 17.3 |
|  | **LS** | 0.2 | 0.0 | 0.2 | 0.2 | 0.0 | 1.4 | 3.9 | 2.3 | 4.1 | 0.0 | 0.0 | 12.4 |
|  | **S** | 1.2 | 0.3 | 0.0 | 0.3 | 0.0 | 1.2 | 5.3 | 7.4 | 24.5 | 0.0 | 0.0 | 40.1 |
|  | **R** | 0.0 | 0.0 | 0.0 | 0.0 | 0.0 | 0.0 | 0.0 | 0.0 | 0.0 | 0.0 | 0.0 | 0.0 |
|  | **ND** | 0.0 | 0.0 | 0.8 | 0.8 | 0.8 | 3.0 | 4.5 | 1.5 | 9.8 | 0.0 | 0.0 | 21.1 |
|  | **SUM** | 1.4 | 0.3 | 1.6 | 1.8 | 1.0 | 10.9 | 23.1 | 17.2 | 42.6 | 0.0 | 0.0 | 100.0 |

**HWSD**

|  |  | **Field Data** | | | | | | | | | | |  |
| --- | --- | --- | --- | --- | --- | --- | --- | --- | --- | --- | --- | --- | --- |
|  |  | **C** | **SiL** | **L** | **CL** | **SC** | **SCL** | **SL** | **LS** | **S** | **R** | **ND** | **SUM** |
| **Map Data** | **C** | 0.0 | 0.0 | 0.0 | 0.0 | 0.0 | 0.0 | 0.0 | 0.0 | 0.0 | 0.0 | 0.0 | 0.0 |
|  | **SiL** | 0.0 | 0.0 | 0.0 | 0.0 | 0.0 | 0.0 | 0.0 | 0.0 | 0.0 | 0.0 | 0.0 | 0.0 |
|  | **L** | 0.0 | 0.0 | 0.1 | 0.0 | 0.1 | 0.6 | 0.8 | 0.6 | 1.0 | 0.0 | 0.0 | 3.3 |
|  | **CL** | 0.0 | 0.0 | 1.0 | 1.0 | 0.0 | 1.5 | 1.5 | 0.5 | 2.0 | 0.0 | 0.0 | 7.5 |
|  | **SC** | 0.0 | 0.0 | 0.0 | 0.0 | 0.0 | 0.0 | 0.0 | 0.0 | 0.0 | 0.0 | 0.0 | 0.0 |
|  | **SCL** | 0.0 | 0.0 | 0.0 | 0.0 | 0.0 | 0.0 | 0.6 | 0.3 | 0.0 | 0.0 | 0.0 | 0.9 |
|  | **SL** | 0.3 | 0.0 | 0.5 | 0.3 | 0.3 | 5.4 | 11.1 | 8.1 | 7.0 | 0.0 | 0.0 | 33.0 |
|  | **LS** | 0.0 | 0.0 | 0.0 | 0.2 | 0.0 | 0.6 | 1.7 | 1.3 | 2.8 | 0.0 | 0.0 | 6.6 |
|  | **S** | 1.4 | 0.4 | 0.0 | 0.4 | 0.0 | 1.4 | 5.6 | 6.3 | 25.6 | 0.0 | 0.0 | 41.1 |
|  | **R** | 0.0 | 0.0 | 0.0 | 0.0 | 0.0 | 0.0 | 0.0 | 0.0 | 0.0 | 0.0 | 0.0 | 0.0 |
|  | **ND** | 0.0 | 0.0 | 0.0 | 0.0 | 0.0 | 0.0 | 0.8 | 0.0 | 6.7 | 0.0 | 0.0 | 7.5 |
|  | **SUM** | 1.7 | 0.4 | 1.7 | 1.8 | 0.4 | 9.5 | 22.1 | 17.2 | 45.2 | 0.0 | 0.0 | 100.0 |

**SoilGrids1km**

|  |  | **Field Data** | | | | | | | | | | |  |
| --- | --- | --- | --- | --- | --- | --- | --- | --- | --- | --- | --- | --- | --- |
|  |  | **C** | **SiL** | **L** | **CL** | **SC** | **SCL** | **SL** | **LS** | **S** | **R** | **ND** | **SUM** |
| **Map Data** | **C** | 0.0 | 0.0 | 0.0 | 0.0 | 0.0 | 0.0 | 0.0 | 0.0 | 0.0 | 0.0 | 0.0 | 0.0 |
|  | **SiL** | 0.0 | 0.0 | 0.0 | 0.0 | 0.0 | 0.0 | 0.0 | 0.0 | 0.0 | 0.0 | 0.0 | 0.0 |
|  | **L** | 0.0 | 0.0 | 0.0 | 0.0 | 0.0 | 0.0 | 0.0 | 0.0 | 0.0 | 0.0 | 0.0 | 0.0 |
|  | **CL** | 0.0 | 0.0 | 0.0 | 0.0 | 0.0 | 0.0 | 0.0 | 0.0 | 0.0 | 0.0 | 0.0 | 0.0 |
|  | **SC** | 0.0 | 0.0 | 0.0 | 0.0 | 0.0 | 0.0 | 0.0 | 0.0 | 0.0 | 0.0 | 0.0 | 0.0 |
|  | **SCL** | 0.5 | 0.0 | 1.3 | 1.1 | 0.5 | 5.9 | 13.7 | 8.9 | 11.5 | 0.0 | 0.0 | 43.5 |
|  | **SL** | 0.3 | 0.3 | 0.0 | 0.3 | 0.0 | 1.8 | 2.3 | 3.0 | 8.0 | 0.0 | 0.0 | 15.8 |
|  | **LS** | 0.8 | 0.0 | 0.0 | 0.0 | 0.0 | 0.8 | 2.8 | 4.3 | 16.6 | 0.0 | 0.0 | 25.3 |
|  | **S** | 0.0 | 0.0 | 0.0 | 0.0 | 0.0 | 0.0 | 0.0 | 0.0 | 0.0 | 0.0 | 0.0 | 0.0 |
|  | **R** | 0.0 | 0.0 | 0.0 | 0.0 | 0.0 | 0.0 | 0.0 | 0.0 | 0.0 | 0.0 | 0.0 | 0.0 |
|  | **ND** | 0.0 | 0.0 | 0.0 | 0.0 | 0.0 | 1.6 | 4.4 | 2.4 | 6.4 | 0.0 | 0.0 | 14.8 |
|  | **SUM** | 1.6 | 0.3 | 1.3 | 1.3 | 0.5 | 10.1 | 23.1 | 18.6 | 42.6 | 0.0 | 0.0 | 99.4 |

**SoilGrids250m**

|  |  | **Field Data** | | | | | | | | | | |  |
| --- | --- | --- | --- | --- | --- | --- | --- | --- | --- | --- | --- | --- | --- |
|  |  | **C** | **SiL** | **L** | **CL** | **SC** | **SCL** | **SL** | **LS** | **S** | **R** | **ND** | **SUM** |
| **Map Data** | **C** | 0.0 | 0.0 | 0.0 | 0.0 | 0.0 | 0.0 | 0.0 | 0.0 | 0.0 | 0.0 | 0.0 | 0.0 |
|  | **SiL** | 0.0 | 0.0 | 0.0 | 0.0 | 0.0 | 0.0 | 0.0 | 0.0 | 0.0 | 0.0 | 0.0 | 0.0 |
|  | **L** | 0.0 | 0.0 | 0.0 | 0.0 | 0.0 | 0.0 | 0.0 | 0.0 | 0.0 | 0.0 | 0.0 | 0.0 |
|  | **CL** | 0.0 | 0.0 | 0.0 | 0.0 | 0.0 | 0.0 | 0.0 | 0.0 | 0.0 | 0.0 | 0.0 | 0.0 |
|  | **SC** | 0.0 | 0.0 | 0.0 | 0.0 | 0.0 | 0.0 | 0.0 | 0.0 | 0.0 | 0.0 | 0.0 | 0.0 |
|  | **SCL** | 0.0 | 0.4 | 0.8 | 0.4 | 0.0 | 0.0 | 2.6 | 1.1 | 2.3 | 0.0 | 0.0 | 7.5 |
|  | **SL** | 0.7 | 0.0 | 0.7 | 1.3 | 0.7 | 9.8 | 18.7 | 9.2 | 12.8 | 0.0 | 0.0 | 53.8 |
|  | **LS** | 0.0 | 0.0 | 0.0 | 0.0 | 0.0 | 1.4 | 4.0 | 7.8 | 24.2 | 0.0 | 0.0 | 37.4 |
|  | **S** | 0.0 | 0.0 | 0.0 | 0.0 | 0.0 | 0.0 | 0.0 | 0.3 | 0.6 | 0.0 | 0.0 | 0.9 |
|  | **R** | 0.0 | 0.0 | 0.0 | 0.0 | 0.0 | 0.0 | 0.0 | 0.0 | 0.0 | 0.0 | 0.0 | 0.0 |
|  | **ND** | 0.0 | 0.0 | 0.0 | 0.0 | 0.0 | 0.0 | 0.0 | 0.0 | 0.0 | 0.0 | 0.0 | 0.0 |
|  | **SUM** | 0.7 | 0.4 | 1.4 | 1.7 | 0.7 | 11.3 | 25.4 | 18.4 | 39.8 | 0.0 | 0.0 | 99.7 |

**WISE30sec**

|  |  | **Field Data** | | | | | | | | | | |  |
| --- | --- | --- | --- | --- | --- | --- | --- | --- | --- | --- | --- | --- | --- |
|  |  | **C** | **SiL** | **L** | **CL** | **SC** | **SCL** | **SL** | **LS** | **S** | **R** | **ND** | **SUM** |
| **Map Data** | **C** | 0.0 | 0.0 | 0.0 | 0.0 | 0.0 | 0.0 | 0.0 | 0.0 | 0.0 | 0.0 | 0.0 | 0.0 |
|  | **SiL** | 0.0 | 0.0 | 0.0 | 0.0 | 0.0 | 0.0 | 0.0 | 0.0 | 0.0 | 0.0 | 0.0 | 0.0 |
|  | **L** | 0.1 | 0.0 | 0.1 | 0.1 | 0.0 | 0.7 | 1.3 | 0.7 | 0.9 | 0.0 | 0.0 | 3.8 |
|  | **CL** | 0.0 | 0.0 | 0.0 | 0.0 | 0.0 | 0.0 | 0.0 | 0.0 | 0.0 | 0.0 | 0.0 | 0.0 |
|  | **SC** | 0.0 | 0.0 | 0.0 | 0.0 | 0.0 | 0.0 | 0.0 | 0.0 | 0.0 | 0.0 | 0.0 | 0.0 |
|  | **SCL** | 0.0 | 0.0 | 0.0 | 0.0 | 0.0 | 0.0 | 0.0 | 0.0 | 0.0 | 0.0 | 0.0 | 0.0 |
|  | **SL** | 0.5 | 0.0 | 1.9 | 0.9 | 0.9 | 7.5 | 18.2 | 11.7 | 15.9 | 0.0 | 0.0 | 57.5 |
|  | **LS** | 0.0 | 0.0 | 0.0 | 0.0 | 0.0 | 0.0 | 0.0 | 0.0 | 0.0 | 0.0 | 0.0 | 0.0 |
|  | **S** | 0.6 | 0.3 | 0.0 | 0.3 | 0.0 | 1.9 | 4.7 | 6.9 | 23.4 | 0.0 | 0.0 | 38.1 |
|  | **R** | 0.0 | 0.0 | 0.0 | 0.0 | 0.0 | 0.0 | 0.0 | 0.0 | 0.0 | 0.0 | 0.0 | 0.0 |
|  | **ND** | 0.0 | 0.0 | 0.0 | 0.0 | 0.0 | 0.0 | 0.0 | 0.0 | 0.0 | 0.0 | 0.0 | 0.0 |
|  | **SUM** | 1.1 | 0.3 | 1.9 | 1.3 | 0.9 | 10.1 | 24.2 | 19.3 | 40.2 | 0.0 | 0.0 | 99.4 |
